# Longitudinal Transcriptomic Remodeling of Adipose Tissue After Bariatric Surgery Revealed by Differential Expression and Explainable Machine Learning

**DOI:** 10.3390/genes17080974

**Published:** 2026-08-19

**Authors:** Soumaya Allouch, Md. Shaheenur Islam Sumon, Aisha Naeem, Claus Vinter Bødker Hviid, Zumin Shi, Muhammad E. H. Chowdhury, Shona Pedersen

**Affiliations:** 1 Department of Basic Medical Science, College of Medicine, QU Health, Qatar University, Doha 2713, Qatar; sallouch@qu.edu.qa; 2 Department of Clinical Medicine, College of Medicine, Aalborg University, 9260 Aalborg, Denmark; 3 Department of Electrical Engineering, Qatar University, Doha 2713, Qatar; sumon@qu.edu.qa; 4 Health Sector, Qatar University, Doha 2713, Qatar; a.naeem@qu.edu.qa; 5 Department of Clinical Biochemistry, Aalborg University Hospital, 9000 Aalborg, Denmark; claus.hviid@rn.dk; 6 College of Health Sciences, QU Health, Qatar University, Doha 2713, Qatar; zumin@qu.edu.qa

**Keywords:** bariatric surgery, white adipose tissue, longitudinal transcriptomics, machine learning, explainable artificial intelligence, metabolic remodeling

## Abstract

**Background:** Bariatric surgery improves metabolic health, but long-term transcriptomic remodeling of white adipose tissue (WAT) after Roux-en-Y gastric bypass (RYGB) remains incompletely defined. This study aimed to characterize longitudinal WAT gene-expression patterns after RYGB and prioritize candidate signatures of post-surgical adaptation using a publicly available dataset. **Methods:** We analyzed subcutaneous WAT transcriptomic data from women with obesity who underwent RYGB, with samples collected before surgery and at 2 and 5 years after surgery. Differential expression analysis was integrated with pathway enrichment, supervised machine-learning-based feature prioritization and classification, and SHAP-based model interpretation. **Results:** Differential expression and machine-learning analyses showed clear separation between baseline and post-surgery transcriptomic states. Pathway-level findings indicated reduced inflammatory and immune-related signaling, particularly across pathways related to phagosome function, lysosomal activity, antigen presentation, and host-defense responses after surgery. Gene-level analyses additionally suggested extracellular-matrix and metabolic remodeling. Machine-learning models distinguished baseline from post-surgery samples, while SHAP analysis identified genes with the strongest contributions to model predictions. Importantly, several statistically prioritized genes also showed high SHAP attribution, demonstrating concordance between univariate statistical significance and multivariate predictive relevance. This convergence suggests that the models captured biologically meaningful surgery-associated signals rather than purely data-driven classification artifacts. **Conclusions:** This study advances the interpretation of longitudinal adipose-tissue transcriptomic remodeling after RYGB by combining differential expression, pathway enrichment, supervised machine learning, and explainable AI within a unified framework. The integrated workflow prioritized candidate long-term remodeling genes, particularly immune/inflammatory and extracellular-matrix-related transcriptomic signatures, that warrant validation in independent cohorts.

## 1. Introduction

Obesity is a chronic, multifactorial disease associated with substantial disturbances in metabolic homeostasis, including insulin resistance, dyslipidemia, type 2 diabetes, and increased cardiovascular risk. Central to the development of these complications is white adipose tissue (WAT), which functions not only as an energy storage site but also as a dynamic endocrine and immune organ. In obesity, WAT undergoes extensive structural and functional remodeling characterized by adipocyte hypertrophy, altered adipokine secretion, immune cell infiltration, and chronic low-grade inflammation, all of which contribute to systemic metabolic dysfunction [1,2].

Roux-en-Y gastric bypass (RYGB) surgery is one of the most effective interventions for severe obesity, producing substantial and sustained weight loss together with marked improvements in insulin sensitivity, lipid metabolism, and glycemic control. Importantly, the metabolic benefits of RYGB often persist long after the period of maximal weight loss and may remain evident even in the presence of partial weight regain [3,4]. These observations indicate that the long-term efficacy of bariatric surgery cannot be explained solely by changes in body weight or fat mass, but instead reflects durable biological adaptations at the tissue and molecular levels.

Growing evidence suggests that adipose tissue plays a central role in mediating the sustained metabolic benefits of bariatric surgery. Transcriptomic profiling of human subcutaneous WAT has revealed extensive gene-expression remodeling following RYGB, involving pathways related to inflammation, immune signaling, lipid metabolism, mitochondrial function, and adipocyte differentiation [5,6]. Notably, although many transcriptional changes occur within the first one to two years after surgery, a subset of genes, particularly those associated with inflammatory and immune processes, continue to show progressive regulation up to five years postoperatively, independent of weight regain [5]. These persistent molecular adaptations may contribute to improved adipose tissue morphology and endocrine function, thereby supporting long-term metabolic health.

Despite these insights, comprehensive longitudinal analyses capturing both intermediate and long-term transcriptomic changes in human adipose tissue remain limited. Many existing studies focus on short-term follow-up or cross-sectional comparisons, which restricts the ability to distinguish transient from sustained molecular responses to surgery. Moreover, given known sex-specific differences in adipose tissue biology and metabolic regulation, there is a particular need for well-characterized longitudinal studies in female cohorts.

High-throughput transcriptomic datasets present substantial analytical challenges due to their high dimensionality, complex correlation structure, and potential redundancy among features. Traditional statistical approaches, such as differential expression analysis, are essential for identifying genes that change significantly between conditions but may not fully capture multivariate expression patterns that optimally distinguish physiological states over time [7]. Machine learning (ML) approaches provide a complementary framework by enabling feature prioritization, classification, and pattern recognition in high-dimensional data, thereby improving the identification of biologically informative gene signatures [7,8].

In recent years, ML methods have been increasingly applied to biomedical and metabolic research, including studies of bariatric surgery outcomes, to enhance predictive performance and uncover complex molecular relationships [9,10]. A related study by Chen et al. combined WGCNA, LASSO, and SVM-RFE across public adipose-tissue datasets after bariatric surgery, identifying STMN2, SFRP4, APOE, and MXRA5 as candidate genes. The present study extends this work by examining 2- and 5-year longitudinal changes after RYGB and applying SHAP to interpret gene contributions to model predictions [11]. More recently, Lee et al. used longitudinal single-nucleus adipose-tissue transcriptomics to characterize cell-type-specific responses after bariatric surgery and identify genetic regulators associated with interindividual variability in weight loss [12]. In contrast, the present study examines longer-term bulk WAT remodeling at 2 and 5 years after RYGB and applies explainable machine learning to prioritize genes distinguishing pre- and post-surgery states. Recent single-nucleus studies have additionally demonstrated cell-type-specific remodeling of brown/beige adipose tissue after bariatric surgery and rapid changes in human subcutaneous WAT during the first postoperative year. These findings complement the present study, which focuses on longer-term bulk WAT transcriptomic changes at 2 and 5 years after RYGB [13]. However, the interpretability of ML models remains a critical concern, particularly in translational research. Explainable artificial intelligence approaches, such as SHapley Additive exPlanations (SHAP), address this limitation by quantifying the contribution of individual features to model predictions, thereby improving transparency and biological interpretability [14,15].

In the present study, we analyzed a publicly available longitudinal transcriptomic dataset of subcutaneous white adipose tissue from women who underwent Roux-en-Y gastric bypass surgery, with samples collected before surgery and specifically at 2 and 5 years after surgery. The central focus of this work was to integrate conventional statistical analysis with supervised machine learning and explainable artificial intelligence to identify gene-expression signatures that are not only differentially expressed but also predictive and interpretable. Specifically, we combined FDR-corrected differential expression analysis, pathway enrichment, ensemble-based feature prioritization, classification modeling, and SHAP-based explainability to prioritize genes that were simultaneously statistically significant, discriminative, and influential in model predictions. This integrated strategy allowed us to move beyond single-method gene ranking and distinguish broadly altered transcriptomic signals from genes with stronger multivariate predictive relevance. Therefore, the main contribution of this study is a unified statistical and explainable machine-learning framework for prioritizing candidate long-term adipose tissue remodeling signatures after bariatric surgery. To our knowledge, this study is among the first to combine intermediate- and long-term longitudinal adipose-tissue transcriptomics after bariatric surgery with differential-expression analysis, ensemble-based feature prioritization, classification, and SHAP-based model interpretation within a unified framework.

## 2. Materials and Methods

### 2.1. Data Source and Study Population

This study utilized a publicly available longitudinal transcriptomic dataset of subcutaneous adipose tissue from women with obesity who underwent Roux-en-Y gastric bypass surgery. The dataset included transcriptomic and clinical data collected before surgery and during postoperative follow-up, as described in the original source study [16]. Abdominal subcutaneous white adipose tissue (WAT) biopsies were obtained before surgery (*n* = 50), 2 years post-surgery (*n* = 49), and 5 years post-surgery (*n* = 38), with 37 women providing samples at all three time points. For long-term comparison, the original study included an age-matched, non-operated comparison group of 28 women with stable body weight. Because all participants in the dataset were women, the comparison group was sex-comparable to the RYGB cohort. However, these controls were not weight- or BMI-matched to the RYGB group and should therefore be described as an age-matched non-operated comparison group rather than as weight-matched or BMI-matched controls. Global gene expression profiling was performed using Affymetrix Clariom™ D microarrays (Affymetrix, Inc., Santa Clara, CA, USA). Alongside transcriptomic data, extensive clinical, metabolic, and adipose phenotyping was collected, including BMI, fat mass (DXA-derived ESAT and EVAT), insulin resistance (HOMA-IR), lipid profiles, adipokines, adipocyte size and number, and lipolysis markers [16]. All participants in the dataset were women. The present analysis included the longitudinal RYGB cohort and the age-matched, non-operated comparison group, which was used as a contextual reference. The third cohort from the original study was not included in the present analysis. Ethical approval was obtained from the local ethics committee in Stockholm, and written informed consent was provided by all participants; no financial compensation was offered. All examinations were performed at 8:00 AM after an overnight fast. Anthropometric measurements and clinical chemistry analyses were conducted according to previously published protocols, and total body fat mass was estimated using bioelectrical impedance analysis [16].

### 2.2. Sample Collection and Preparation

The gene expression analyses were based on white adipose tissue (WAT) obtained by needle biopsy from abdominal subcutaneous fat, collected lateral to the umbilicus at each sampling time point. Immediately after collection, biopsies were rinsed in a 9 mg/mL sodium chloride solution and divided into two portions: approximately 300 mg of tissue was snap-frozen in liquid nitrogen and stored for subsequent gene expression profiling, while the remaining fraction was used for adipocyte isolation to assess fat cell size as part of the phenotypic characterization [16].

### 2.3. Gene Expression Data Source and Preprocessing

The present study used publicly available adipose tissue transcriptomic data generated in the original source study [16]. In that study, global gene-expression profiling of subcutaneous white adipose tissue was performed using Affymetrix Clariom™ D microarrays. For the present analysis, we used the available processed gene-expression data and mapped probe-set identifiers to HGNC-approved gene symbols. All probe-set identifiers were mapped to HGNC-approved gene symbols, and obsolete gene symbols, transcript suffixes, and platform-specific probe annotations were replaced with current HGNC nomenclature where applicable. The publicly available GEO metadata for GSE199063 were reviewed for potential technical variables, including processing batch, scan date, hybridization date, scanner identity, and array plate or processing run. The sample records reported a common hybridization protocol, use of an Affymetrix GeneChip Scanner 3000 7G (Affymetrix, Inc., Santa Clara, CA, USA), and preprocessing with Affymetrix Expression Console version 1.4.1 using the SST-RMA method. However, explicit sample-level processing-batch, scan-date, hybridization-date, scanner-serial-number, and array plate/run variables were not provided. Consequently, these technical factors could not be directly incorporated as covariates. Residual confounding by unrecorded technical variation therefore cannot be completely excluded. No new RNA extraction, microarray profiling, lncRNA-specific analysis, Qlucore Omics Explorer analysis, or experimental qRT-PCR validation was performed in the present study. Any qRT-PCR validation mentioned in this manuscript refers only to validation reported in the original source study.

### 2.4. Statistical Analysis

Gene expression differences between time points were evaluated using paired *t*-tests for within-subject comparisons, specifically comparing post-surgery samples with pre-surgery (baseline) samples at 2- and 5-year follow-ups. Because attained age increased systematically with follow-up time, chronological age and longitudinal timepoint were intrinsically linked. Therefore, the present dataset did not permit independent estimation of age-related transcriptomic drift and surgery-associated temporal changes by including both attained age and timepoint as separate covariates. The longitudinal comparisons should consequently be interpreted as identifying time point-associated changes observed after RYGB rather than effects attributable exclusively to surgery.

Samples were matched across timepoints using participant identifiers, yielding 49 matched pairs for baseline versus 2 years and 38 matched pairs for baseline versus 5 years; missing follow-up samples were excluded pairwise, with no imputation performed. Among the 37 participants with expression measurements available at all three timepoints, within-participant changes were calculated as postoperative minus baseline expression. Directional concordance between the 2- and 5-year changes was summarized as the proportion of participants showing changes in the same direction, and Spearman correlation was used to assess similarity between the corresponding participant-level changes. Results were reported as *p*-values with the direction of change. To account for multiple testing and reduce the risk of false positives, *p*-values were adjusted using the Benjamini–Hochberg false discovery rate (FDR) method [17], and genes with FDR ≤ 0.05 were considered statistically significant [18]. For volcano-plot classification, genes were considered upregulated or downregulated when FDR ≤ 0.05 and |log_2_FC| ≥ 1; genes meeting only the FDR criterion were not included in the displayed upregulated and downregulated counts. Exploratory and presentation analyses included principal component analysis (PCA) [19] to visualize global transcriptomic separation across groups/time points [20], volcano plots [21] to jointly display effect size and statistical significance, and box plots to illustrate group-wise distributions for representative genes. The non-operated comparison group was used only as a contextual reference for baseline transcriptomic differences. To determine whether the observed separation between pre- and post-surgery samples was dependent on differential-expression-based gene selection, an additional unsupervised PCA was performed using all approximately 25,500 quality-controlled annotated genes before FDR-based gene filtering. Separate all-gene PCA analyses were conducted for baseline versus 2 years post-surgery and baseline versus 5 years post-surgery. These analyses were used as a sensitivity assessment of the global transcriptomic structure rather than as formal evidence for the absence of batch effects. Partial least squares discriminant analysis (PLS-DA) was performed in R version 4.4.2 using the mixOmics package, installed through BiocManager (version 1.30.27), to explore supervised separation between baseline and postoperative samples and identify genes contributing to group discrimination. The primary analyses in the present study focused on longitudinal within-cohort comparisons between baseline and post-surgery RYGB samples at 2 and 5 years. Because the non-operated comparison group was age-matched but not weight- or BMI-matched to the RYGB cohort, baseline-versus-control transcriptomic differences may reflect clinical differences in adiposity, insulin resistance, adipocyte morphology, and obesity-associated inflammation rather than surgery-related effects. Therefore, baseline-versus-control findings were interpreted cautiously and were not used as the main basis for surgery-related conclusions.

### 2.5. Machine Learning Analysis

Machine-learning (ML) models were employed to classify and analyze the data following a structured data-partitioning, preprocessing, feature-selection, and modeling pipeline. To account for the longitudinal study design and prevent participant-level data leakage, the participant-disjoint train/test split was performed before any standardization, feature ranking, feature selection, or model fitting. Samples were partitioned using participant identifiers, and all samples collected from the same woman across the baseline, 2-year, and 5-year timepoints were assigned to the same partition. Therefore, no participant contributed samples to both the training and test datasets. The data were divided into a participant-disjoint training set (80%) and a held-out test set (20%). To enhance training efficiency and ensure that all features contributed comparably, the Standard Scaler [22] was fitted exclusively using the training data and subsequently applied to the held-out test data using the scaling parameters estimated from the training partition. Given the high dimensionality of the dataset, feature ranking was applied as a strategy to reduce overfitting. Feature importance was computed using three tree-based feature-selection models—Random Forest [23], XGBoost [24], and ExtraTrees [25]—fitted exclusively using the training partition. The hyperparameters of the three tree-based feature-selection models were evaluated during preliminary tuning using the training partition, and the best-performing configurations were retained for the final feature-ranking analysis. XGBoost (2.1.1) was configured with a maximum depth of 4, a learning rate of 0.2, an L2 regularization parameter of 1, 150 estimators, and subsample and feature-subsampling ratios of 0.9. Random Forest was configured with 50 estimators and a maximum tree depth of 10. ExtraTrees was fitted using the selected configuration reported in Supplementary Table S2. Fixed random seeds were used to ensure reproducibility. The complete model configurations are provided in Supplementary Table S2, whereas Supplementary Table S4 describes the final Random Forest classifier used for outcome classification. The importance scores obtained from the three models were normalized and averaged to reduce model-specific bias and identify features consistently prioritized across the different ensemble architectures. The final top-20 genes were selected from this training-derived combined importance ranking; the held-out test samples were not used to determine the reported gene list.

The top-20 ranked genes constituted the initial candidate feature pool rather than the fixed feature set used in every final classifier. Within the participant-disjoint training partition, classifiers were evaluated using subsets of the ranked genes, and the number of features producing the best classification performance was selected. The final best-performing model used the top seven ranked genes for the baseline-versus-2-year comparison and the top three ranked genes for the baseline-versus-5-year comparison. SHAP values were calculated using these final classifiers and the exact feature sets included in each model. Consequently, the SHAP plots display all features used by the corresponding final classifier rather than a truncated subset of a 20-feature model. Five-fold cross-validation was performed within the training set using participant-grouped folds, ensuring that all observations from a given participant remained within a single fold. Using the training-derived selected features, eight classifiers were trained and evaluated: Linear Discriminant Analysis, Logistic Regression, Random Forest, ExtraTrees, XGBoost, LightGBM [26], AdaBoost [27], and Gradient Boosting [28]. After model comparison, the final classifier was fitted using the complete training partition and evaluated once on the untouched held-out test set. The held-out test data were not used during standardization, feature ranking, feature selection, classifier comparison, or parameter tuning. The training-derived candidate genes were subsequently used for pathway enrichment, SHAP-based interpretation, expression visualization, and single-gene ROC analysis. These downstream analyses did not contribute to feature selection, classifier development, or calculation of the multigene held-out test performance reported in the Results section All ML-selected genes, together with their corresponding ML importance scores, log_2_FC values, and FDR-adjusted *p*-values, are provided in Supplementary Table S1. Evaluation metrics, including accuracy, precision, recall, specificity, and F1-score, were computed, and the corresponding equations are provided in Supplementary Section S1.

### 2.6. Gene Set Enrichment Analysis

To identify biological pathways remodeled after surgery, Kyoto Encyclopedia of Genes and Genomes (KEGG) pathway enrichment analysis was performed. The top-ranking genes, selected based on adjusted *p*-values, were analyzed at two time points—2 years and 5 years. Enrichment results were compared between two gene selection strategies: a bioinformatics approach based on differential expression analysis and a machine learning–based approach. The gene set used for the machine-learning-based enrichment analysis was derived exclusively from the participant-disjoint training partition. The held-out test samples were not used to rank genes or define the machine-learning gene list submitted for pathway enrichment analysis. Pathways were considered significantly enriched if the false discovery rate (FDR)–adjusted *p*-value was less than 0.05.

**Figure 1:**
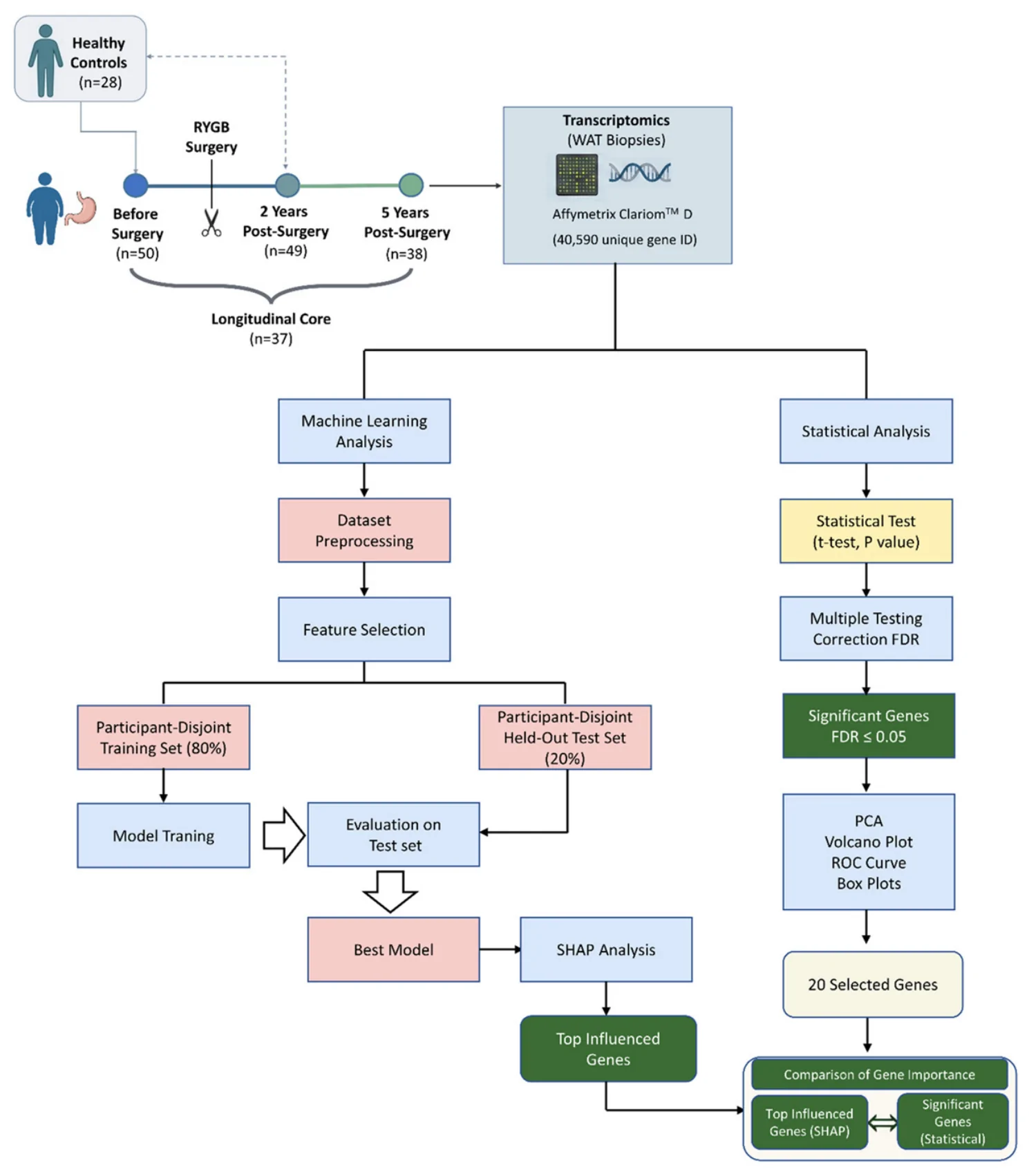
Overview of the statistical and machine-learning workflow for gene expression analysis following bariatric surgery.

The overall workflow of the study is shown in Figure 1. After defining the study cohorts and timepoints, the analysis proceeds through two complementary branches: machine learning and statistical testing. In the machine learning branch, the data are preprocessed, informative features are selected, and models are trained using a train/test split, followed by test-set evaluation to identify the best-performing model. Model interpretability is then performed using Shapley (version 0.46.0) Additive explanation (SHAP) [29] to determine the most influential genes driving predictions. In the statistical analysis branch, group-wise testing is carried out, followed by multiple-testing correction (FDR) to control false discoveries and identify significant genes. Key visual outputs (e.g., PCA, volcano, ROC, and box plots) summarize expression patterns and discrimination performance. Finally, results from both branches are compared to highlight genes that are consistently supported by both statistical significance and ML-based importance.

## 3. Results

### 3.1. Characteristics of Study Populations

Table 1 summarizes key demographic, anthropometric, and metabolic parameters measured at baseline and at 2 and 5 years after RYGB surgery. Values are reported as mean ± standard deviation for each follow-up timepoint. Changes from baseline to 2 years and from baseline to 5 years are evaluated using the corresponding *p*-values shown in the last two columns. The results indicate marked improvements in body weight, BMI, total fat mass, and insulin resistance indices after surgery. Glycemic control metrics (glucose, insulin, HOMA-IR) show significant reductions, reflecting improved metabolic status. Lipid-related measures also shift favorably, including lower triglycerides and higher HDL cholesterol over time. Overall, the table highlights sustained post-surgical benefits, with some attenuation between 2 and 5 years for select measures. Mean age increased from 43 ± 9 years at baseline to 45 ± 9 years at 2 years and 47 ± 10 years at 5 years. This increase reflects the expected passage of time within the same longitudinal cohort rather than a baseline imbalance between independent study groups. Nevertheless, because age and follow-up time progress together, age-related changes in adipose-tissue gene expression cannot be fully separated from the transcriptomic changes observed after surgery. Mean age increased from 43 ± 9 years at baseline to 45 ± 9 years at 2 years and 47 ± 10 years at 5 years. This increase reflects the expected passage of time within the same longitudinal cohort rather than a baseline imbalance between independent study groups. Nevertheless, because age and follow-up time progress together, age-related changes in adipose-tissue gene expression cannot be fully separated from the transcriptomic changes observed after surgery. In addition to systemic metabolic improvements, adipose tissue phenotyping showed a marked reduction in fat cell volume after RYGB. Fat cell volume decreased from 970 ± 185 pL at baseline to 466 ± 174 pL at 2 years and 458 ± 168 pL at 5 years after surgery, indicating sustained improvement in adipocyte morphology. However, formal histological measures such as fibrosis staining, crown-like structures, vascular density, or immune-cell infiltration were not available in the public dataset and therefore could not be analyzed.

### 3.2. Differential Expression Analysis and Machine Learning Results

**Figure 2:**
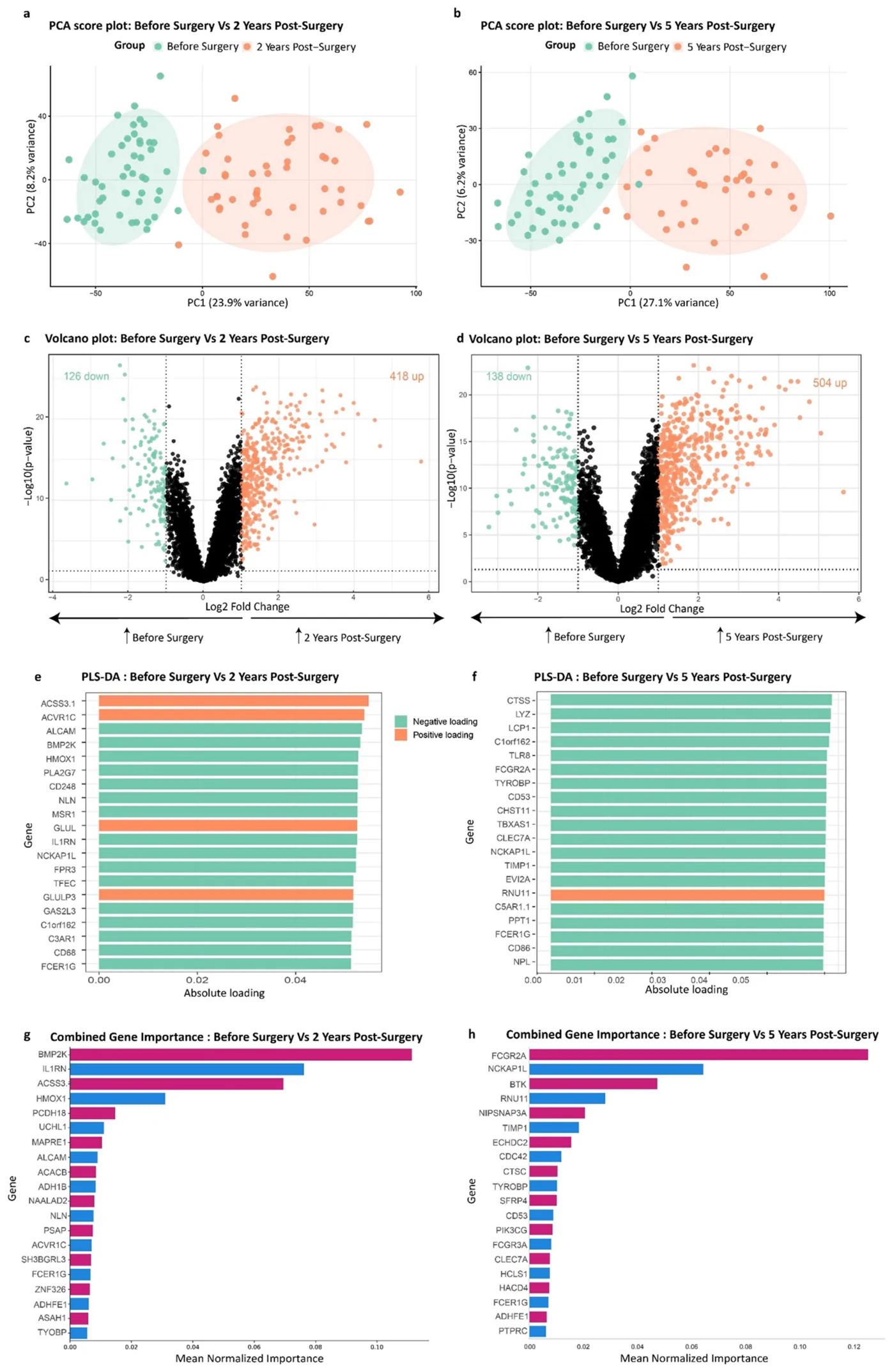
Global transcriptomic remodeling following RYGB surgery. (**a**,**b**) Principal component analysis (PCA) score plots showing separation between baseline and post-surgery samples for before surgery vs. 2 years post-surgery (**a**) and before surgery vs. 5 years post-surgery (**b**). (**c**,**d**) Volcano plots showing differentially expressed genes for before surgery vs. 2 years post-surgery (**c**) and before surgery vs. 5 years post-surgery (**d**). The x-axis represents log2 fold change, and the y-axis represents −log10(*p*-value); significantly upregulated and downregulated genes are highlighted. (**e**,**f**) PLS-DA loading plots showing the genes contributing most strongly to group separation for before surgery vs. 2 years post-surgery (**e**) and before surgery vs. 5 years post-surgery (**f**). (**g**,**h**) Combined machine-learning gene-importance rankings based on averaged feature-importance scores from tree-based models for before surgery vs. 2 years post-surgery (**g**) and before surgery vs. 5 years post-surgery (**h**).

In the statistical analysis workflow, group-wise differential expression analysis was performed, followed by false discovery rate (FDR) correction to control for multiple testing. After quality control and filtering, including the exclusion of unannotated or unidentified probe sets, the final analysis was carried out on approximately 25,000 annotated genes selected from the original 40,590 transcript features. Genes with an FDR ≤ 0.05 were considered statistically significant and were subsequently used for downstream visualization and candidate gene prioritization. Following FDR correction, 6825 significant genes were identified in the comparison between before surgery and 2 years post-surgery, while 6479 significant genes were identified in the comparison between before surgery and 5 years post-surgery. In Supplementary Figure S1, a Venn diagram summarizes the overlap of significant DE genes across the three longitudinal comparisons. A total of 3030 genes were common to all three comparisons. In addition, 510 genes were shared only between baseline versus control and baseline versus 2 years post-surgery, and 337 genes were shared only between baseline versus control and baseline versus 5 years post-surgery. The two post-surgery comparisons shared 1528 genes, while the numbers of unique significant genes were 644 for baseline versus control, 1757 for baseline versus 2 years post-surgery, and 1584 for baseline versus 5 years post-surgery. These results indicate substantial overlap in the transcriptomic changes detected across the longitudinal statistical comparisons. Figure 2a,b presents PCA score plots computed using the statistically significant genes (FDR ≤ 0.05). In both panels, samples from Before Surgery form a distinct cluster that separates from 2 Years Post-Surgery and 5 Years Post-Surgery, indicating a clear shift in global gene-expression patterns after surgery. The separation is primarily driven by PC1, suggesting that the major variance reflects differences between the pre-surgery and post-surgery states. Overall, the PCA results confirm that the selected significant genes effectively distinguish Before Surgery from long-term follow-up timepoints. Figure 2c,d shows the volcano plots for differential expression comparing Before Surgery vs. 2 Years Post-Surgery and Before Surgery vs. 5 Years Post-Surgery. The x-axis represents log2 fold change, while the y-axis shows −log10(*p*), so genes farther from the center and higher on the plot indicate stronger and more significant changes. In the 2 Years Post-Surgery comparison, 418 genes are upregulated, and 126 are downregulated relative to Before Surgery. In the 5 Years Post-Surgery comparison, the number of changes is slightly larger, with 504 upregulated and 138 downregulated genes. Overall, both panels indicate a predominance of upregulated genes after surgery and demonstrate sustained transcriptomic remodeling at both follow-up timepoints. In Supplementary Figure S2, the volcano plot comparing 2-year and 5-year post-surgery samples shows that 4 genes are significantly upregulated and 23 genes are significantly downregulated between the two timepoints. To assess whether the observed group separation depended on prior selection of statistically significant genes, we additionally performed unsupervised PCA using all approximately 25,500 quality-controlled annotated genes before differential-expression-based filtering. As shown in Supplementary Figure S3, baseline and post-surgery samples demonstrated broad separation along PC1 in both comparisons. PC1 accounted for 8.1% of the total variance in the baseline versus 2-year comparison and 9.0% in the baseline versus 5-year comparison, while PC2 accounted for 6.7% and 5.5%, respectively. Some overlap between the groups remained, particularly near the center of the PCA space. These findings indicate that the global pre- and post-surgery transcriptomic separation was not solely attributable to selecting FDR-significant genes. However, because explicit sample-level batch and array-processing variables were unavailable, this analysis cannot completely exclude residual technical confounding associated with timepoint.

Figure 2e,f shows the PLS-DA loading plots highlighting the genes that contribute most to separating the groups in each comparison. For Before Surgery vs. 2 Years Post-Surgery (Figure 2e) and Before Surgery vs. 5 Years Post-Surgery (Figure 2f), genes with larger absolute loadings have a stronger influence on the discrimination between timepoints. In the PLS-DA Before Surgery vs. 2 Years Post-Surgery, the strongest positive loadings are observed for *ACSS3* and *ACVR1C*, followed by *GLUL*, indicating that these genes contribute most strongly to the separation toward the post-surgery group at 2 years and are likely associated with metabolic adaptation after surgery. In contrast, for Before Surgery vs. 5 Years Post-surgery, *RNU11* stands out as the dominant positively loaded gene, suggesting a distinct long-term molecular signature compared with the earlier post-surgical phase. The common gene between both analyses is *C1orf162*, which appears in both.

Before preprocessing and feature selection, participants were divided into participant-disjoint training and held-out test partitions. The combined gene-importance rankings shown in Figure 2g,h were generated exclusively from the training partition using Random Forest, XGBoost, and ExtraTrees. The final top-20 genes therefore represent a training-derived candidate gene set, and no held-out test samples were used to calculate their feature-importance scores or determine their inclusion in the reported list. In the machine-learning analysis, the top 20 features (genes) were selected using a combined importance score derived from three tree-based models: XGBoost, ExtraTrees, and Random Forest. Figure 2g,h illustrates the resulting mean normalized importance rankings for the before-surgery comparisons with 2 Years Post-Surgery and 5 Years Post-Surgery, respectively. These panels highlight the genes that most consistently contributed to classification performance across the ensemble feature-importance approach. In the Before Surgery vs. 2 Years Post-Surgery model, *BMP2K* emerges as the most important feature, followed by *IL1RN, ACSS3*, and *HMOX1*, highlighting the dominance of metabolic regulation and inflammation-related pathways shortly after surgery. In contrast, the Before Surgery vs. 5 Years Post-surgery model is strongly driven by immune signaling genes, with *FCGR2A* showing the highest importance, followed by *NCKAP1L, BTK*, and *RNU11*, indicating a shift toward immune and leukocyte-associated processes in long-term outcomes.

Using the top features from Figure 2g,h, we trained ML models and summarized the top 5 in Table 2. Random Forest performed best for both comparisons, achieving 97.97% (Before vs. 2 years) and 97.72% (Before vs. 5 years) accuracy/F1. For 2 years, LGBM and AdaBoost also showed high performance (~97% and 96%). For 5 years, LDA was the second-best model (96.59%). Overall, the selected features enable strong separation of post-surgery samples from baseline. The performance metrics reported in Table 2 were calculated using the untouched participant-disjoint held-out test set. This test partition was not used during standardization, feature ranking, feature selection, classifier comparison, or model tuning. Thus, the reported test-set performance was evaluated independently of the feature-selection procedure. The performance of the best-performing Random Forest model was further assessed using confusion matrices and five-fold cross-validated ROC curves (Supplementary Figures S6 and S7). Only a small number of samples were misclassified in the before-surgery versus 2-year and before-surgery versus 5-year comparisons. The ROC curves showed excellent discriminatory performance, with mean AUC values close to 1.00 for both comparisons.

**Figure 3:**
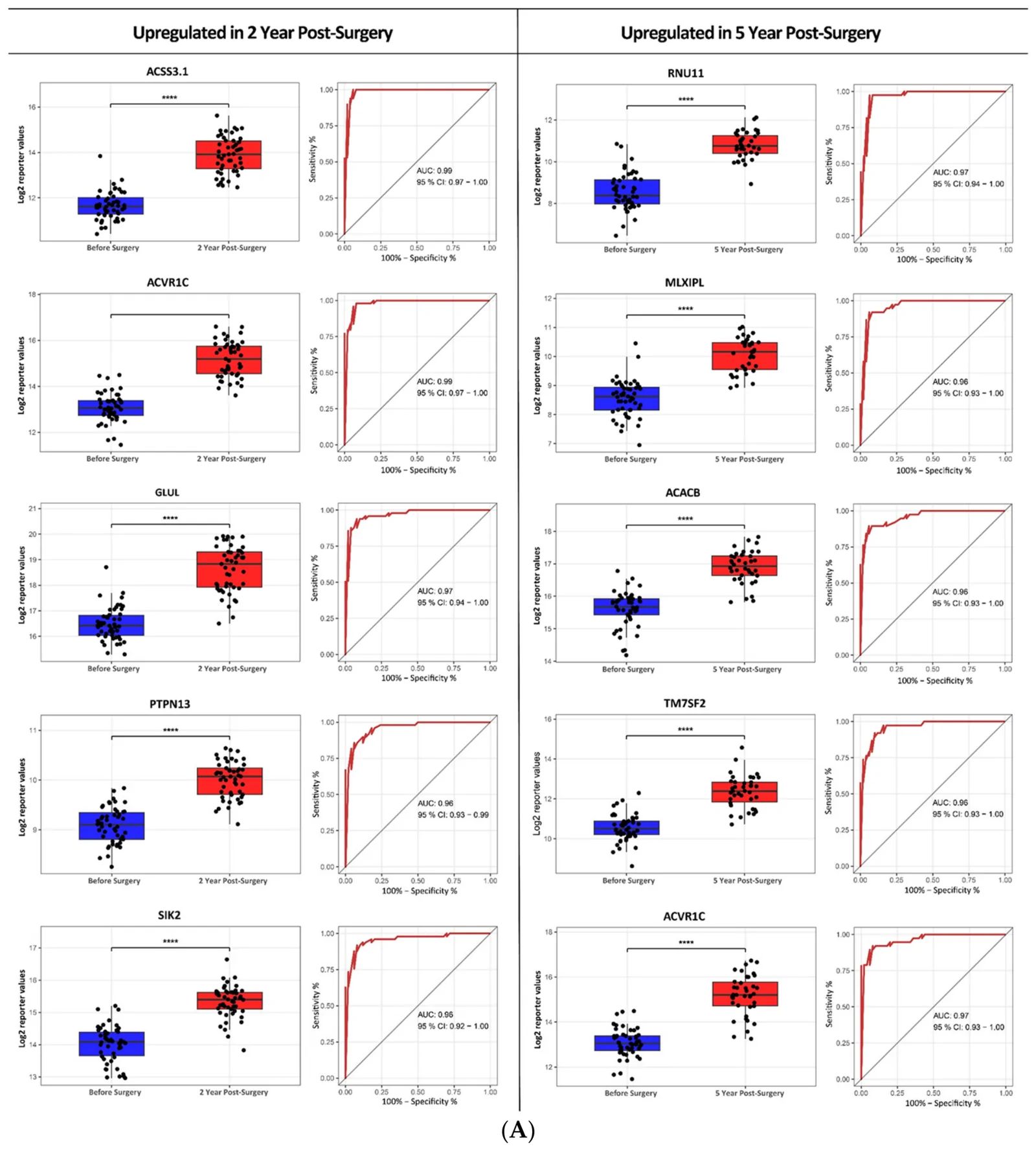

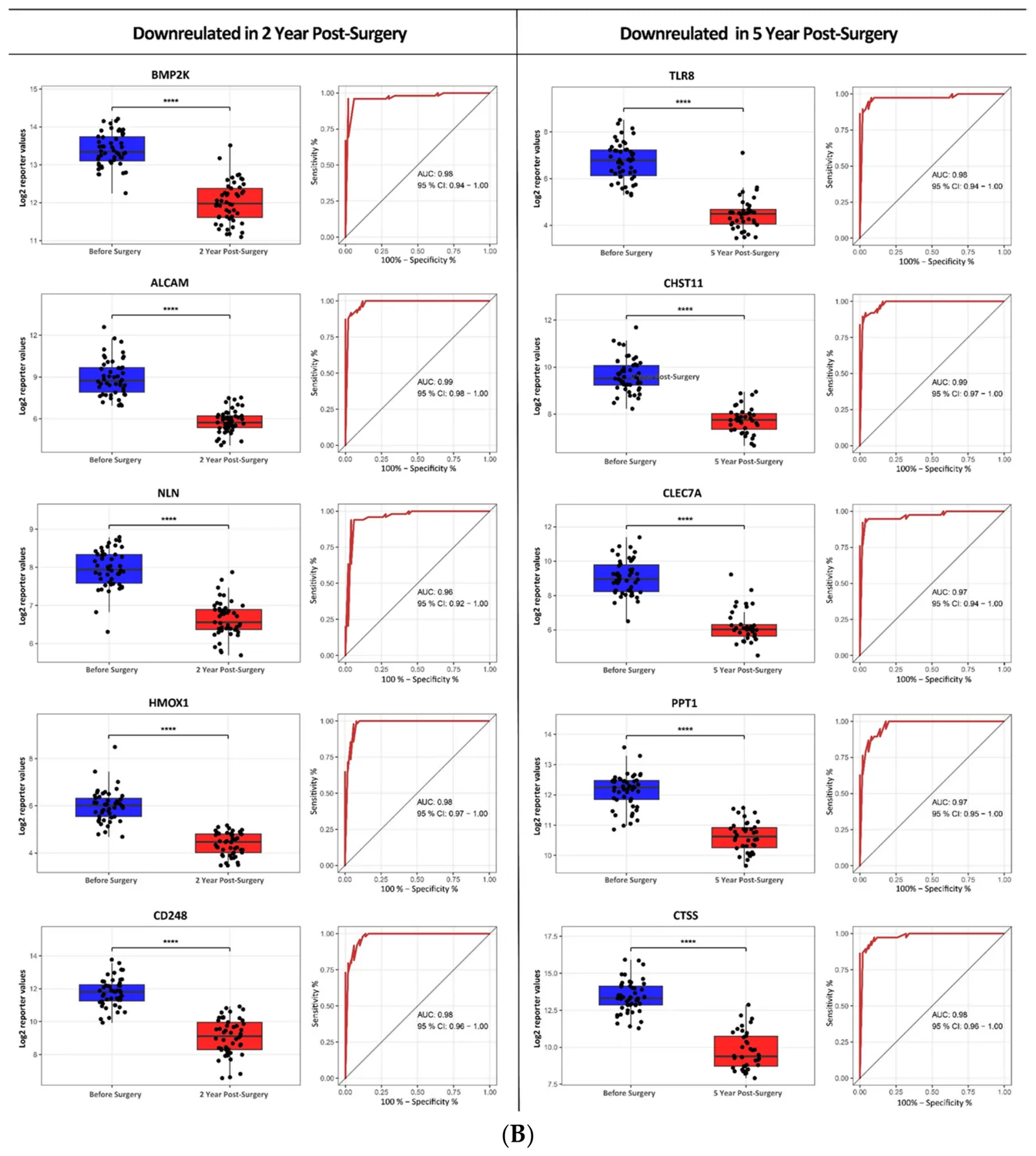
The figure displays data for a selection of the most significantly (**A**) upregulated and (**B**) downregulated genes. For each gene, boxplots (**left**) show normalized expression for baseline (Pre, n = 50), 2-year (2Y, n = 49), and 5-year (5Y, n = 38) groups. Boxes denote the interquartile range (IQR) and whiskers 1.5× IQR. Statistical significance was determined by a paired *t*-test with FDR correction (****, *p* < 0.0001). Corresponding ROC curves (**right**) illustrate each gene’s individual classification performance, with the Area Under the Curve (AUC) noted.

To visualize the expression patterns and individual predictive performance of the most significant genes from this signature, we plotted the top upregulated and downregulated genes in Figure 3A,B, respectively. These figures illustrate the expression patterns of selected genes and show their differential expression trends following surgery based on the re-analyzed transcriptomic dataset. To provide a unified view of the predictive and statistical relevance of each ML-selected gene, we compiled an integrated summary of ML importance scores, log_2_ fold changes, and FDR values (Supplementary Table S1). The complete gene-level ROC curves are presented in Supplementary Figure S4 for baseline versus 2 years post-surgery and Supplementary Figure S5 for baseline versus 5 years post-surgery. As shown in Supplementary Table S1, several genes prioritized at both follow-up timepoints demonstrated consistent group-level expression directions. *FCER1G* and *TYROBP* were downregulated at both 2 and 5 years, whereas *ADHFE1* was upregulated at both timepoints. Nevertheless, these summary fold-change estimates do not indicate the proportion of individual participants showing the same direction of change. To address this, we examined within-participant directional concordance and trajectory consistency for *ALCAM*, *MSR1*, *PLA2G7*, and *NPL* among the 37 participants with expression data at all three timepoints (Supplementary Table S3). The large majority of participants (94.6–100%) showed changes in the same direction as the group-level effect at both 2 and 5 years, and each participant’s 2-year and 5-year changes were strongly correlated (Spearman ρ = 0.83–0.86), indicating that these represent consistent, reproducible individual-level trajectories rather than population-average effects.

### 3.3. Pathway Enrichment Analysis

**Figure 4:**
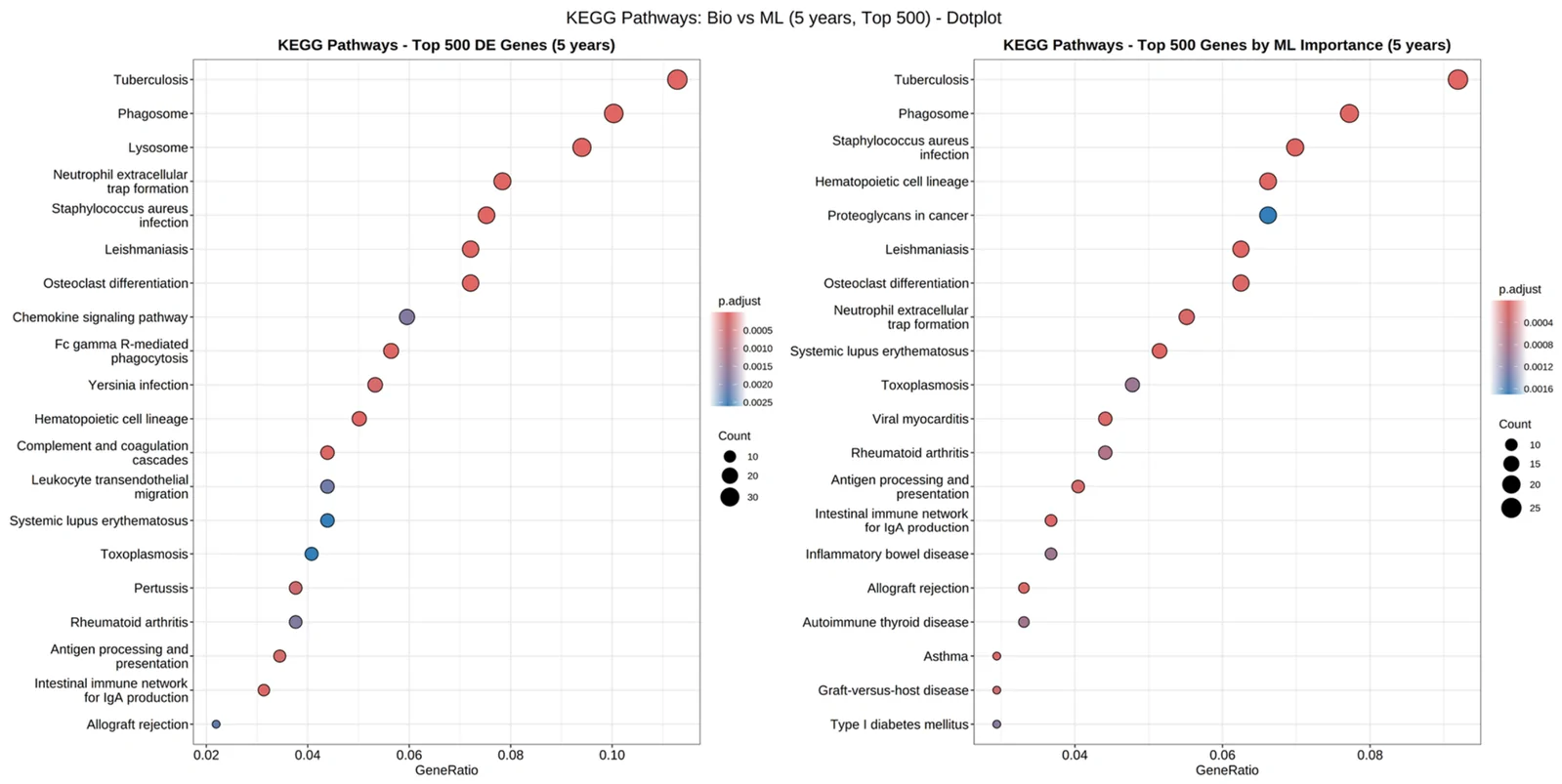
Concordance of KEGG pathway enrichment between analytical methods. The dot plot contrasts the top KEGG pathways derived from the top-ranking genes at 5 years post-surgery. Pathways were identified using two gene-selection strategies: a bioinformatics approach based on differential-expression analysis (“Bio”, blue) and a machine-learning–based approach (“ML”, red). The x-axis represents GeneRatio, dot size indicates pathway gene count, and dot color represents adjusted *p*-value. The substantial overlap of immune- and inflammation-related pathways demonstrates strong concordance in biological interpretation between differential-expression analysis and machine-learning–based gene prioritization.

Having identified and visualized these key predictive genes, we next sought to validate the broader biological themes they represent. We performed a comparative pathway enrichment analysis to test whether our machine learning approach and the standard statistical analysis converged on similar biological functions. The results, shown in Figure 4, reveal a high degree of concordance between the two analytical techniques.

Specifically, several immune- and inflammation-related KEGG pathways were consistently enriched by both the differential-expression and machine-learning approaches. The strongest overlapping pathways included Tuberculosis, Phagosome, Lysosome, Staphylococcus aureus infection, Leishmaniasis, Systemic lupus erythematosus, Rheumatoid arthritis, Antigen processing and presentation, and Intestinal immune network for IgA production. Additional enrichment of Chemokine signaling, Fc gamma R-mediated phagocytosis, Leukocyte transendothelial migration, Complement and coagulation cascades, and Neutrophil extracellular trap formation further supports extensive remodeling of innate and adaptive immune pathways following bariatric surgery. The high degree of overlap between the two analytical approaches indicates that the identified biological signals are robust and not dependent on a single gene-prioritization strategy. It should be noted that many of the enriched KEGG categories—including Tuberculosis, Leishmaniasis, Staphylococcus aureus infection, Systemic lupus erythematosus, and Rheumatoid arthritis—are disease-annotated pathways that largely share a common set of core immune, phagocytic, and antigen-presentation genes. Their enrichment therefore reflects overlapping immune-gene membership across KEGG’s disease categories rather than evidence of disease-specific biological processes occurring in adipose tissue after RYGB. The concordant enrichment patterns identify candidate biological themes associated with the longitudinal transcriptomic changes after RYGB. However, pathway enrichment reflects the overrepresentation of pathway-associated transcripts and does not directly demonstrate altered pathway activity, immune-cell composition, tissue architecture, protein abundance, or functional biological mechanisms. These results should therefore be interpreted as hypothesis-generating transcriptomic associations requiring orthogonal experimental validation.

**Figure 5:**
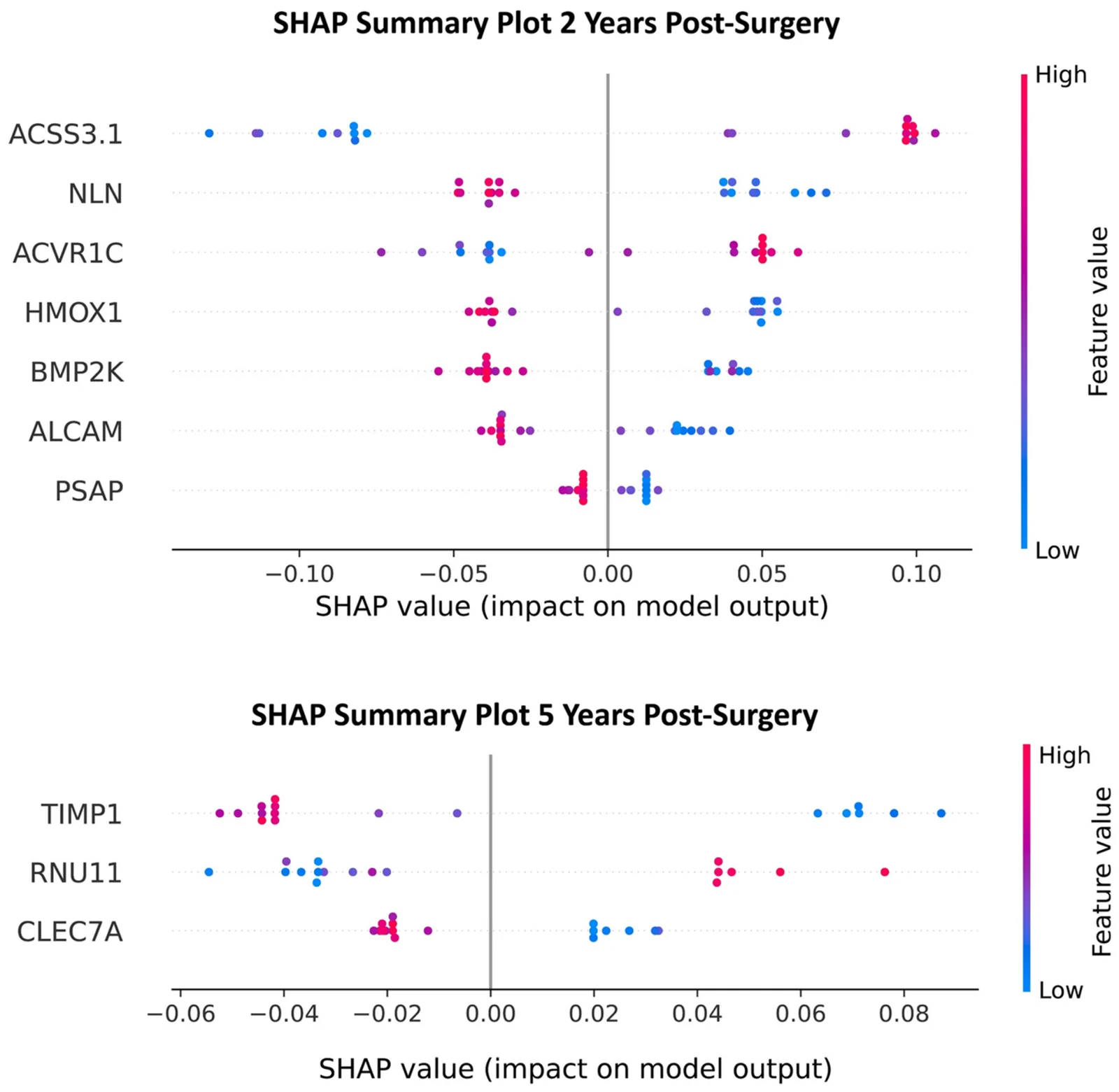
SHAP-based interpretation of the final best-performing classifiers. The baseline-versus-2-year classifier included the seven top-ranked features that produced the best classification performance, whereas the baseline-versus-5-year classifier included the optimal three-feature subset. SHAP values were calculated using these final models and their exact input feature sets. Accordingly, the plots display all features included in each classifier rather than selected top-N features from a 20-gene model. Positive SHAP values shift predictions toward the post-surgery class, whereas negative values shift predictions toward the baseline class.

Although Figure 2g,h presents the 20 highest-ranked candidate genes, the optimal classifiers used smaller subsets of this ranked pool. The best-performing baseline-versus-2-year model included seven genes, whereas the best-performing baseline-versus-5-year model included three genes. SHAP analysis was performed on these final models using the same seven- and three-gene feature sets, respectively. Therefore, all features included in each final classifier are displayed in Figure 5. Finally, to directly link the predictive features of our model to their biological impact, we applied SHapley Additive exPlanations (SHAP). This analysis quantifies how much each gene contributes to the model’s prediction for a sample being pre- or post-surgery. As illustrated in Figure 5, higher gene expression values (red) with positive SHAP scores increased the probability of classifying samples as post-surgery, while lower expression values (blue) with negative SHAP scores contributed toward baseline classification. SHAP analysis showed that statistically prioritized genes, including *RNU11* and *CLEC7A*, also contributed strongly to the corresponding model predictions. This agreement provides model-level interpretability and indicates partial consistency across the statistical and machine-learning analyses. However, SHAP attribution reflects the behavior of the fitted classifier and should not be interpreted as independent confirmation of biological function, particularly for *RNU11*, whose dominant signal may partly reflect microarray-platform characteristics.

## 4. Discussion

Bariatric surgery remains one of the most effective interventions for achieving sustained weight loss and improving metabolic health in individuals with severe obesity. Yet, the biological mechanisms that underpin these long-term benefits, particularly within white adipose tissue (WAT), are yet to be unraveled [4]. As a metabolically active and immunologically responsive organ, WAT undergoes profound remodeling in response to weight loss, including changes in adipocyte function, immune cell composition, and gene expression [1,2]. Recent longitudinal studies have highlighted that these molecular adaptations can persist for years after surgery, suggesting a form of metabolic memory that extends beyond the initial weight loss phase [5,30,31]. In this study, we sought to better understand the temporal dynamics of WAT remodeling by analyzing gene expression profiles in subcutaneous adipose tissue collected before and at two and five years after Roux-en-Y gastric bypass (RYGB). By combining traditional differential expression analysis with machine learning and explainable artificial intelligence (XAI) techniques, we identified distinct early and late gene signatures, as well as a core set of stable transcriptomic signatures that may reflect durable biological reprogramming. These findings offer new insights into the long-term molecular trajectory of adipose tissue following bariatric surgery and highlight the value of interpretable computational tools in uncovering clinically meaningful biomarkers.

Using a publicly available longitudinal transcriptomic dataset, the present study proposes an integrative statistical and explainable machine-learning framework to investigate adipose tissue remodeling after Roux-en-Y gastric bypass surgery. Rather than relying only on conventional differential expression analysis, the proposed pipeline combines FDR-corrected statistical testing, pathway-level interpretation, supervised machine-learning-based feature prioritization, classification, and SHAP-based model explanation. Through this framework, we evaluated whether machine-learning models could distinguish baseline samples from 2-year and 5-year post-surgery samples and identify the genes that contributed most strongly to this separation. The results showed that ML-selected genes were able to discriminate pre- and post-surgery transcriptomic states with high classification performance, while SHAP analysis provided an interpretable ranking of the genes driving model predictions. By integrating statistical significance, fold-change direction, ML feature importance, and SHAP attribution, the proposed method prioritized candidate genes consistently supported across complementary analytical layers, including long-term discriminative features such as RNU11. Therefore, the main contribution of this study is the development and application of a unified interpretable computational pipeline for identifying statistically significant, predictive, and biologically relevant transcriptomic signatures associated with long-term adipose tissue remodeling after bariatric surgery. Importantly, the objective of this study was not to reproduce the findings of the original dataset publication, but rather to determine whether explainable machine-learning approaches could provide complementary biological insights beyond conventional differential-expression analysis. By integrating statistical significance, machine-learning feature importance, and SHAP-based interpretability, we sought to identify transcriptomic signatures that may be overlooked when genes are prioritized solely on the basis of differential expression. The statistical and machine-learning approaches provided partially overlapping but non-identical gene prioritizations, which is expected because they capture different properties of the data. Differential-expression analysis identifies genes with strong univariate changes, whereas ML-based feature importance reflects multivariable discriminative contribution. Nevertheless, overlap across analytical layers was observed for several genes, including RNU11, CLEC7A, ALCAM, ACVR1C, and C1orf162. In addition, the DE analysis showed substantial overlap across the longitudinal comparisons themselves, with 3030 genes shared across all three comparisons and 1528 genes shared between the 2-year and 5-year post-surgery contrasts (Supplementary Figure S1), supporting the existence of a robust shared biological remodeling signal.

A major finding of our study is the profound and sustained suppression of immune and inflammatory pathways within adipose tissue five years after surgery. The downregulation of key genes such as TLR8, a sensor for viral single-stranded RNA, CLEC7A (Dectin-1), a pattern recognition receptor crucial for fungal recognition, and CTSS (Cathepsin S), an enzyme essential for antigen presentation by MHC class II molecules, collectively points to a deep and lasting latency of the resident immune cell populations [32]. In the obese state, adipose tissue is characterized by a chronic, low-grade inflammation driven by stressed adipocytes and pro-inflammatory macrophages, a state directly linked to the development of insulin resistance [33,34]. The coordinated downregulation of these specific genes suggests a reduction in inflammatory gene-expression signatures associated with this state, involving a reduction in both innate immune sensing and the capacity for antigen presentation, which would limit T-cell activation. Our KEGG pathway enrichment analysis identified strong concordance between the statistical and machine-learning approaches, particularly across pathways associated with phagosome biology, lysosomal activity, antigen presentation, chemokine signaling, and host-defense responses (Figure 4). This pathway-level agreement provides robust support for the observed suppression and remodeling of immune-related transcriptional programs following bariatric surgery. More importantly, the durability of this anti-inflammatory signature, persisting even with partial weight regain as noted in other longitudinal studies [30,35], suggests persistent alterations in adipose tissue immune-related transcriptional profiles, rather than simply responding to reduced adipocyte size. This lasting immunomodulation is likely one potential contributor to the long-term improvements in systemic insulin sensitivity and overall metabolic health. Complementing the profound suppression of inflammation, several differentially expressed genes suggested ongoing remodeling of extracellular matrix and cell–matrix interaction processes together with alterations in metabolic programming. In obesity, adipose tissue ECM often becomes excessively cross-linked and fibrotic, impairing healthy adipocyte expansion and promoting a pro-inflammatory environment [36]. The persistent alteration of genes like ACVR1C and ALCAM at both timepoints (Table 3) suggests a durable change in cell-cell and cell-matrix interactions that counteracts this pathological state. Notably, ALCAM, MSR1, PLA2G7, and NPL were independently prioritized as top discriminative features at both the 2-year and 5-year post-surgery timepoints, each with consistent downregulation and AUC values ≥ 0.96 at both intervals. Because these genes emerged from separate comparisons rather than a single longitudinal model, their reproducibility across two independent timepoints strengthens the case that they represent stable, durable transcriptomic markers of postoperative adipose remodeling rather than transient or timepoint-specific signals. This is further supported by within-participant analysis, which showed near-complete directional concordance and strong correlation between each participant’s 2- and 5-year expression changes (Spearman ρ = 0.83–0.86; Supplementary Table S3). Our comparative KEGG pathway analysis complements these findings by identifying highly concordant enrichment of pathways related to phagosome function, lysosomal activity, antigen processing and presentation, and immune-cell signaling (Figure 4). Together, these findings suggest that long-term adipose tissue remodeling after bariatric surgery is characterized not only by changes in metabolic pathways but also by sustained alterations in immune-cell activity and inflammatory regulation.

This may reflect persistent remodeling of the tissue architecture away from a fibrotic state towards a more flexible and healthier scaffold, which is critical for restoring proper adipocyte function and insulin sensitivity [37]. Furthermore, the emergence of a distinct 5-year signature, marked by the upregulation of transcription factors like MLXIPL (carbohydrate-responsive element-binding protein), a master regulator of de novo lipogenesis [38] points towards a sophisticated, long-term adaptation in metabolic programming. This suggests that after an initial phase of rapid catabolism, the tissue establishes a new homeostatic set point, actively regulating lipid metabolism in a manner that likely contributes to the sustained maintenance of metabolic improvements [39].

A key strength and novel contribution of our study is the integration of explainable AI (XAI) to move beyond prediction and into biological interpretation. While machine learning models are powerful, their “black box” nature can limit their translational value [14]. By applying SHAP analysis, we were able to deconstruct our model’s decisions on a per-gene, per-sample basis, confirming that the model’s predictions were driven by biologically plausible features. The SHAP summary plots (Figure 5) provide a striking visualization of this, showing, for instance, that higher expression of the upregulated gene RNU11 consistently pushed the model’s prediction toward a “post-surgery” classification. Conversely, higher expression of the pro-inflammatory gene CLEC7A drove the prediction toward “baseline.” This demonstrates a tight coupling between a gene’s statistical significance (Figure 3A,B), its predictive importance for classification (Table 3), and its interpretable contribution to the model’s output. This multi-layered agreement increases confidence that the prioritized genes reflect biologically relevant post-surgical remodeling signals. Importantly, although genes such as ALCAM, MSR1, PLA2G7, and NPL reliably distinguished baseline from post-surgical transcriptomic states, the present analysis does not establish whether these genes predict the magnitude of individual clinical outcomes such as improvements in insulin sensitivity, HOMA-IR, or weight-loss trajectories. Future studies integrating gene-expression and clinical outcome data will be necessary to evaluate their prognostic utility.

It should be emphasized that SHAP values explain how the fitted model uses gene expression to classify samples; they do not establish gene function, causal relevance, or biomarker validity on their own. Agreement between SHAP importance and differential expression reflects consistency in statistical signal detection rather than independent biological validation. However, these findings should be considered candidate transcriptomic signatures that require validation in independent cohorts and experimental studies before their potential biomarker utility can be established. More broadly, our integrative framework serves as a powerful template for future longitudinal studies, illustrating how explainable AI can be used to transform complex transcriptomic data into testable hypotheses with clear clinical and biological relevance [15,40].

RNU11, a core component of the minor (U12-dependent) spliceosome, emerged as a dominant long-term classifier in our analysis. Although small nuclear RNAs (snRNAs) are not typically highlighted in adipose-tissue transcriptomics [41], their expression can reflect broader changes in cellular activity, including altered splicing demands in immune cells or shifts in adipocyte turnover following weight-loss surgery. At present, the biological significance of RNU11 upregulation remains unclear, and we cannot exclude the possibility that its strong signal partly reflects platform-specific characteristics of microarrays, which often detect snRNAs with high sensitivity and dynamic range. Nonetheless, its consistent importance across statistical and machine-learning approaches suggests that changes in the minor spliceosome may accompany long-term adipose remodeling. Further studies using RNA-seq or targeted snRNA quantification will be necessary to determine whether RNU11 represents a true biological signature or a technical artifact.

While this study provides a comprehensive longitudinal and interpretable analysis of adipose tissue transcriptomic remodeling after RYGB, several considerations should be noted when interpreting the findings. First, the study cohort consisted exclusively of women, which reduces biological heterogeneity but may limit the generalizability of the results to male populations. A further limitation concerns the potential influence of aging on the longitudinal transcriptomic differences. Participants were approximately two and five years older at the respective postoperative follow-ups, and chronological aging may independently affect adipose-tissue gene expression. Because attained age and time since surgery increased together within participants, their effects were intrinsically confounded and could not be separated using the available dataset. Thus, the ML classifiers and SHAP attributions may reflect a combination of surgery-associated remodeling, aging-related expression drift, and other time-dependent biological changes. The age-matched non-operated group was used only as a contextual reference and did not provide a longitudinal, BMI-matched control trajectory sufficient to isolate aging effects. Attrition from 50 baseline participants to 38 participants at 5 years may have introduced selection bias. Because baseline characteristics could not be formally compared between participants with and without long-term samples, the 5-year findings may preferentially represent participants who remained in follow-up. Future studies should evaluate attrition-related differences and use appropriate methods to account for missing longitudinal observations. A longitudinal non-operated comparison cohort sampled over equivalent intervals would be required to distinguish age-related changes from those more specifically associated with RYGB. In addition, long-term transcriptomic changes at the 5-year timepoint could be influenced by age-related processes and hormonal transitions such as menopause, factors known to independently alter adipose tissue biology, especially in female populations [42,43]. Second, analyses were confined to subcutaneous white adipose tissue and therefore may not fully capture molecular remodeling processes occurring in visceral adipose depots, which are also critically involved in metabolic risk and inflammation [2,44]. Although fat cell volume was available and showed sustained reduction after RYGB, detailed histological assessment of adipose tissue was not available in the public dataset. Therefore, we could not directly evaluate tissue fibrosis, crown-like structures, vascular density, or immune-cell infiltration. As a result, interpretations regarding extracellular matrix remodeling and inflammatory changes are based on transcriptomic and pathway-level findings rather than direct histological validation. Because bulk WAT contains multiple cell populations, reduced immune-related gene expression may reflect altered cellular composition rather than transcriptional reprogramming within specific cell types. Third, gene expression profiling was performed using microarray technology, which offers robust longitudinal comparability but may have reduced sensitivity for low-abundance transcripts or novel isoforms compared with RNA-sequencing approaches [45]. A further limitation concerns the interpretation of the high single-gene AUCs and classification performance. Because this study re-analyzed a publicly available microarray dataset, explicit sample-level information regarding processing batch, scan date, hybridization date, scanner serial number, and array plate or processing run was unavailable. Therefore, these technical factors could not be incorporated directly as covariates, and residual confounding between longitudinal timepoint and unrecorded technical variation cannot be completely excluded. The additional PCA based on all quality-controlled genes demonstrated that the separation between baseline and post-surgery samples was not solely caused by differential-expression-based feature selection. However, unsupervised PCA cannot distinguish a biological timepoint effect from a technical factor that is strongly or completely confounded with timepoint. Accordingly, the high AUCs should be interpreted as evidence of strong within-dataset discrimination rather than externally validated biomarker performance. A further important limitation is the lack of external validation: although the dataset was divided into training and testing subsets, all analyses—differential expression, feature selection, and classification—were conducted using a single publicly available cohort, and the training and test partitions therefore share the same underlying population, processing pipeline, and technical conditions. Consequently, the genes identified here (including ALCAM, MSR1, PLA2G7, NPL, BMP2K, IL1RN, FCGR2A, and others highlighted in Table 3) should be regarded as candidate biomarkers of post-surgical adipose remodeling rather than definitive, externally validated molecular signatures. Additionally, feature stability across alternative train-test splits and resampling iterations was not assessed. Given the high dimensionality of the dataset (~25,000 genes) relative to the modest number of samples per comparison (<100), the top-ranked genes identified from a single train/test partition may be sensitive to sampling variation. Future work should evaluate gene-ranking stability using repeated resampling or bootstrap-based feature selection to confirm the robustness of the reported gene rankings. Moreover, concordance between the differential-expression and machine-learning analyses does not constitute independent validation, as both approaches were applied to the same expression matrix and time-point labels; agreement between the two reflects consistency in signal detection within a single dataset rather than confirmation from an independent data source. Independent validation in a separately collected and processed longitudinal cohort, and/or experimental verification (e.g., targeted qPCR or protein-level confirmation), would substantially strengthen confidence in these findings and remains necessary before clinical or biomarker application can be considered. Additionally, individual-level associations between weight trajectory and gene-expression changes were not analyzed. Although Table 1 suggests partial weight regain at the group level between 2 and 5 years after RYGB, we did not correlate individual changes in weight or BMI with inflammatory gene signatures. Therefore, claims regarding transcriptomic persistence despite weight regain were avoided. More broadly, the present study demonstrates that ALCAM, MSR1, PLA2G7, and NPL reliably distinguish pre- from post-surgical timepoints, but does not establish whether the magnitude of their expression change predicts individual clinical outcomes, such as degree of insulin sensitivity improvement or metabolic recovery. Testing this relationship using HOMA-IR, insulin, and body-composition measures available in this cohort represents an important direction for future analysis. Furthermore, the identified transcriptomic signatures were not evaluated in an independent external cohort. Future studies should prioritize replication of these candidate genes in an independently collected and processed RYGB cohort, ideally profiled with RNA-sequencing, and should pursue orthogonal experimental validation (e.g., qPCR or protein-level assays) to confirm that the ML- and SHAP-prioritized genes reflect reproducible biological signals rather than dataset-specific artifacts.

Finally, although the paired longitudinal design strengthens inference and reduces inter-individual variability, the observational nature of the study precludes causal conclusions regarding the functional roles of individual genes. Future studies integrating multi-omic profiling, functional validation, and more diverse cohorts will be important to further elucidate the mechanisms underlying long-term adipose tissue remodeling after bariatric surgery. In addition to the technical and age-related confounds discussed above, information on concomitant medications, diabetes status, and other comorbidities was not available for this cohort and could therefore not be evaluated as potential covariates influencing the observed transcriptomic changes. Future studies with richer clinical metadata will be needed to determine the extent to which treatment-related and comorbidity-related factors contribute to, or are independent of, the long-term adipose tissue changes reported here.

## 5. Conclusions

In summary, this study provides a comprehensive and interpretable analysis of long-term white adipose tissue transcriptomic remodeling following Roux-en-Y gastric bypass surgery. By integrating longitudinal differential expression analysis with machine learning-based feature prioritization and SHAP-driven model interpretability, we identified distinct early and late molecular signatures that reflect dynamic metabolic adaptation and sustained suppression and remodeling of immune-related pathways, including phagosome, lysosomal, antigen-presentation, and inflammatory signaling networks. Importantly, the strong concordance between statistical significance, classification performance, and explainable-AI attribution highlights a subset of stable transcriptomic signatures associated with long-term adipose tissue remodeling that warrant further validation in independent cohorts. Our findings support the concept that the long-term metabolic benefits of bariatric surgery are underpinned by persistent molecular changes in adipose tissue that extend beyond weight loss alone. More broadly, our integrative analytical framework demonstrates how combining transcriptomics with interpretable machine learning can enhance biological insight in longitudinal metabolic studies and may inform future efforts to identify biomarkers and therapeutic targets for sustained metabolic health following obesity interventions.

## Figures and Tables

**Table 1:** Clinical and Metabolic Characteristics at Baseline and During Follow-up After RYGB Surgery.

	Baseline (*n* = 50)	2 Years Post-Surgery (*n* = 49)	5 Years Post-Surgery (*n* = 38)	0 vs. 2 y’s (*p*)	0 vs. 5 y’s (*p*)
Age (years)	43 ± 9	45 ± 9	47 ± 10	<0.0001	<0.0001
Weight (kg)	118 ± 15	80 ± 14	88 ± 18	<0.0001	<0.0001
Body mass index (kg/m^2^)	43 ± 5	29 ± 4	32 ± 6	<0.0001	<0.0001
HOMA-IR (units)	4.0 ± 2.6	1.0 ± 0.4	1.3 ± 0.6	<0.0001	<0.0001
S-Insulin (mU/L)	15.5 ± 8.4	4.9 ± 1.9	5.8 ± 2.7	<0.0001	<0.0001
P-Glucose (mmol/L)	5.6 ± 1.4	4.9 ± 0.6	5.0 ± 0.5	<0.0001	0.0051
Total fat mass (kg)	61 ± 10	31 ± 11	38 ± 14	<0.0001	<0.0001
Fat cell volume (pL)	970 ± 185	466 ± 174	458 ± 168	<0.0001	<0.0001
P-Triglycerides (mmol/L)	1.5 ± 0.7	0.9 ± 0.4	0.9 ± 0.3	<0.0001	<0.0001
P-Cholesterol (mmol/L)	4.8 ± 1.0	4.0 ± 0.8	4.2 ± 0.7	<0.0001	<0.0001
P-HDL cholesterol (mmol/L)	1.2 ± 0.3	1.5 ± 0.3	1.6 ± 0.5	<0.0001	<0.0001

**Table 2:** Performance metrics of the top five machine-learning models.

Before Surgery vs. After 2 Years						Before Surgery vs. After 5 Years					
Models	A	P	R	S	F1	Models	A	P	R	S	F1
Random Forest	97.97	98.05	97.97	97.93	97.97	Random Forest	97.72	97.72	97.72	97.64	97.72
LGBM	96.96	97.14	96.96	97.03	96.96	LDA	96.59	96.62	96.59	96.77	96.59
AdaBoost	95.95	96.03	95.95	95.91	95.95	AdaBoost	93.18	93.31	93.18	93.55	93.19
Gradient Boosting	92.92	92.94	92.92	92.94	92.92	Gradient Boosting	90.90	91.83	90.9	92.45	90.95
XGB	89.89	90.16	89.89	89.97	89.88	LGBM	90.9	90.9	90.9	90.56	90.9

Abbreviations: A, accuracy; P, precision; R, recall; S, specificity; F1, F1-score.

**Table 3:** Top discriminative genes ranked by adjusted *p*-value (FDR) and AUC.

Before Surgery vs. 2 Years Post-Surgery						Before Surgery vs. 5 Years Post-Surgery					
Gene Name	Full Form	log_2_FC	Adj *p* Value	AUC	95% CI	Gene Name	Full Form	log_2_FC	Adj *p* Value	AUC	95% CI
*ACSS3*	Acyl-CoA synthetase short chain family member 3	2.22	6.71 × 10^−23^	0.98	(0.97–1.00)	*RNU11*	RNA, U11 small nuclear	2.24	1.35 × 10^−19^	0.97	(0.94–1.00)
*ACVR1C*	Activin A receptor type 1C	2.09	4.66 × 10^−22^	0.98	(0.97–1.00)	*TLR8*	Toll-like receptor 8	−2.25	1.35 × 10^−19^	0.97	(0.94–1.00)
*BMP2K*	BMP2 inducible kinase	−1.39	1.07 × 10^−20^	0.97	(0.94–1.00)	*CHST11*	Carbohydrate sulfotransferase 11	−1.88	1.35 × 10^−19^	0.98	(0.97–1.00)
*ALCAM*	Activated leukocyte cell adhesion molecule	−3.15	1.58 × 10^−20^	0.99	(0.98–1.00)	*CLEC7A*	C-type lectin domain containing 7A	−2.82	6.24 × 10^−19^	0.97	(0.94–1.00)
*NLN*	Neurolysin	−1.31	1.58 × 10^−20^	0.96	(0.92–1.00)	*PPT1*	Palmitoyl-protein thioesterase 1	−1.51	7.37 × 10^−19^	0.97	(0.95–1.00)
*HMOX1*	Heme oxygenase 1	−1.6	3.11 × 10^−20^	0.98	(0.97–1.00)	*CTSS*	Cathepsin S	−3.64	7.37 × 10^−19^	0.98	(0.96–1.00)
*CD248*	CD248 molecule (endosialin)	−2.69	3.58 × 10^−20^	0.98	(0.96–1.00)	*C1orf162*	Chromosome 1 open reading frame 162	−3.01	1.06 × 10^−18^	0.97	(0.95–1.00)
*NCKAP1L*	NCK-associated protein 1 like	−2.32	3.58 × 10^−20^	0.97	(0.95–1.00)	*IL1RN*	Interleukin 1 receptor antagonist	−4.3	1.06 × 10^−18^	0.97	(0.96–1.00)
*MSR1*	Macrophage scavenger receptor 1	−3.63	3.63 × 10^−20^	0.97	(0.96–1.00)	*PLA2G7*	Phospholipase A2 group VII	−4.48	1.06 × 10^−18^	0.97	(0.94–1.00)
*PSAP*	Prosaposin	−0.93	8.75 × 10^−20^	0.97	(0.95–1.00)	*TIMP1*	TIMP metallopeptidase inhibitor 1	−2.38	1.09 × 10^−18^	0.99	(0.98–1.00)
*GLUL*	Glutamate-ammonia ligase (glutamine synthetase)	2.13	8.87 × 10^−20^	0.96	(0.94–1.00)	*C5AR1*	Complement C5a receptor 1	−2.70207	1.30 × 10^−18^	0.97	(0.95–1.00)
*FPR3*	Formyl peptide receptor 3	−3.17	1.56 × 10^−19^	0.97	(0.95–1.00)	*ALCAM*	Activated leukocyte cell adhesion molecule	−3.29	2.72 × 10^−18^	0.97	(0.95–1.00)
*C1orf162*	Chromosome 1 open reading frame 162	−2.55677	1.74 × 10^−19^	0.97	(0.94–1.00)	*MSR1*	Macrophage scavenger receptor 1	−4	2.72 × 10^−18^	0.96	(0.94–1.00)
*TFEC*	Transcription factor EC	−2.04106	1.74 × 10^−19^	0.96	(0.93–0.99)	*LCP1*	Lymphocyte cytosolic protein 1	−4.16	4.79 × 10^−18^	0.97	(0.95–1.00)
*GAS2L3*	Growth arrest specific 2 like 3	−1.68174	1.74 × 10^−19^	0.97	(0.95–1.00)	*NPL*	N-acetylneuraminate pyruvate lyase	−2.62	5.20 × 10^−18^	0.98	(0.96–1.00)
*PLA2G7*	Phospholipase A2 group VII	−3.98	1.78 × 10^−19^	0.98	(0.96–1.00)	*VAMP8*	Vesicle associated membrane protein 8	−1.95256	1.54 × 10^−17^	0.97	(0.94–1.00)
*CD68*	CD68 molecule	−2.31	2.68 × 10^−19^	0.97	(0.95–1.00)	*LAPTM5*	Lysosomal protein transmembrane 5	−2.59584	2.08 × 10^−17^	0.97	(0.95–1.00)
*CXCL16*	C-X-C motif chemokine ligand 16	−2.45	3.78 × 10^−19^	0.97	(0.94–1.00)	*ARPC5*	Actin-related protein 2/3 complex subunit 5	−1.10349	2.08 × 10^−17^	0.96	(0.93–0.99)
*PTPN13*	Protein tyrosine phosphatase non-receptor type 13	0.92	3.97 × 10^−19^	0.96	(0.93–0.99)	*CD86*	CD86 molecule	−1.99893	2.08 × 10^−17^	0.97	(0.95–1.00)
*NPL*	N-acetylneuraminate pyruvate lyase	−2.21	4.32 × 10^−19^	0.97	(0.95–1.00)	*IL18*	Interleukin 18	−1.82347	3.23 × 10^−17^	0.95	(0.92–1.00)

## Data Availability

The data analyzed in this study are publicly available from the original source dataset/repository reported by Kerr et al. [13]. No new experimental data were generated in the present study.

## Supplementary Materials

The following supporting information can be downloaded at: https://www.mdpi.com/article/10.3390/genes17080974/s1, Figure S1. Venn diagram showing the overlap of significant differentially expressed (DE) genes among three comparisons; Figure S2. Volcano plot of differential gene expression between 2-year and 5-year post-surgery; Figure S3. All-gene principal component analysis of transcriptomic profiles before surgery and after surgery. (A) PCA comparison between before-surgery and 5-year post-surgery samples. (B) PCA comparison between before-surgery and 2-year post-surgery samples; Figure S4. Receiver operating characteristic (ROC) curves of the top discriminative genes distinguishing before-surgery and 2-year post-surgery samples; Figure S5. Receiver operating characteristic (ROC) curves of the top discriminative genes distinguishing before-surgery and 5-year post-surgery samples; Figure S6. Confusion matrices for the best-performing model (Random Forest) classifying (A) Before Surgery vs. 2 Years Post-Surgery and (B) Before Surgery vs. 5 Years Post-Surgery; Figure S7. ROC curves (5-fold cross-validation) for the best-performing model (Random Forest) classifying (A) Before Surgery vs. 2 Years Post-Surgery and (B) Before Surgery vs. 5 Years Post-Surgery; Section S1. Evaluation Metrics; Table S1. Integrated Machine Learning Feature Importance and Statistical Significance of Differentially Expressed Genes for Top Ranked Genes; Table S2. Best-performing hyperparameter configurations of the tree-based models used for feature selection; Table S3. Within-participant directional concordance and trajectory correlation of ALCAM, MSR1, PLA2G7, and NPL expression changes from baseline to 2 and 5 years post-surgery (*n* = 37 participants with complete longitudinal data). All Spearman correlations were statistically significant (*p* < 0.0001); Table S4. Hyperparameter configuration of the final best-performing Random Forest classifier.

## Abbreviations

The following abbreviations are used in this manuscript:

Abbreviation	Full Term
AI	Artificial Intelligence
AUC	Area Under the Curve
BMI	Body Mass Index
CI	Confidence Interval
DEG	Differentially Expressed Gene
ECM	Extracellular Matrix
FDR	False Discovery Rate
GO	Gene Ontology
GSEA	Gene Set Enrichment Analysis
HOMA-IR	Homeostatic Model Assessment of Insulin Resistance
IFNG	Interferon Gamma
IL	Interleukin
KEGG	Kyoto Encyclopedia of Genes and Genomes
LASSO	Least Absolute Shrinkage and Selection Operator
ML	Machine Learning
NK	Natural Killer
PC	Principal Component
PCA	Principal Component Analysis
qRT-PCR	Quantitative Reverse Transcription Polymerase Chain Reaction
RF	Random Forest
ROC	Receiver Operating Characteristic
SHAP	SHapley Additive exPlanations
SST-RMA	Signal Space Transformation–Robust Multi-array Average
TGF-β	Transforming Growth Factor Beta
TNF	Tumor Necrosis Factor
WAT	White Adipose Tissue
