# Supplementary material for: Longitudinal Transcriptomic Remodeling of Adipose Tissue After Bariatric Surgery Revealed by Differential Expression and Explainable Machine Learning
Source: Genes (Basel). 2026 Aug 19;17(8):974. doi: 10.3390/genes17080974 (PMC13511782; doi:10.3390/genes17080974)
Supplement: Supplementary file 1 [file genes-17-00974-s001.zip › genes-4450288-supplementary.pdf]

Supplementary Materials

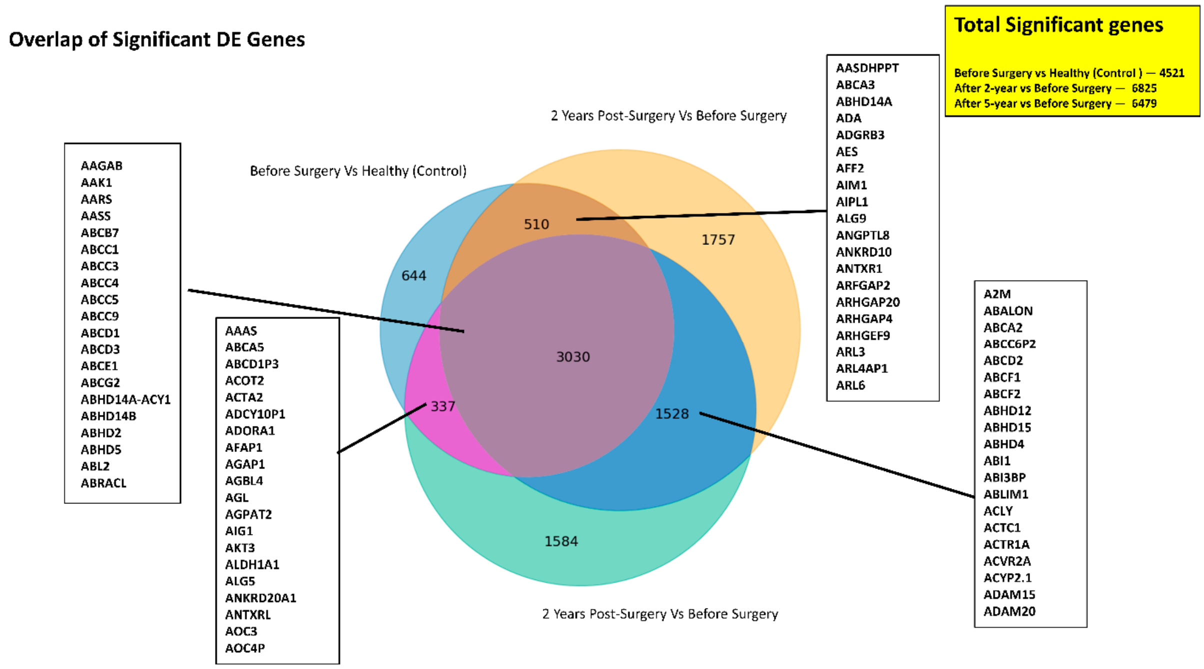

Supplementary Figure S1. Venn diagram showing the overlap of significant differentially expressed (DE) genes among three comparisons.

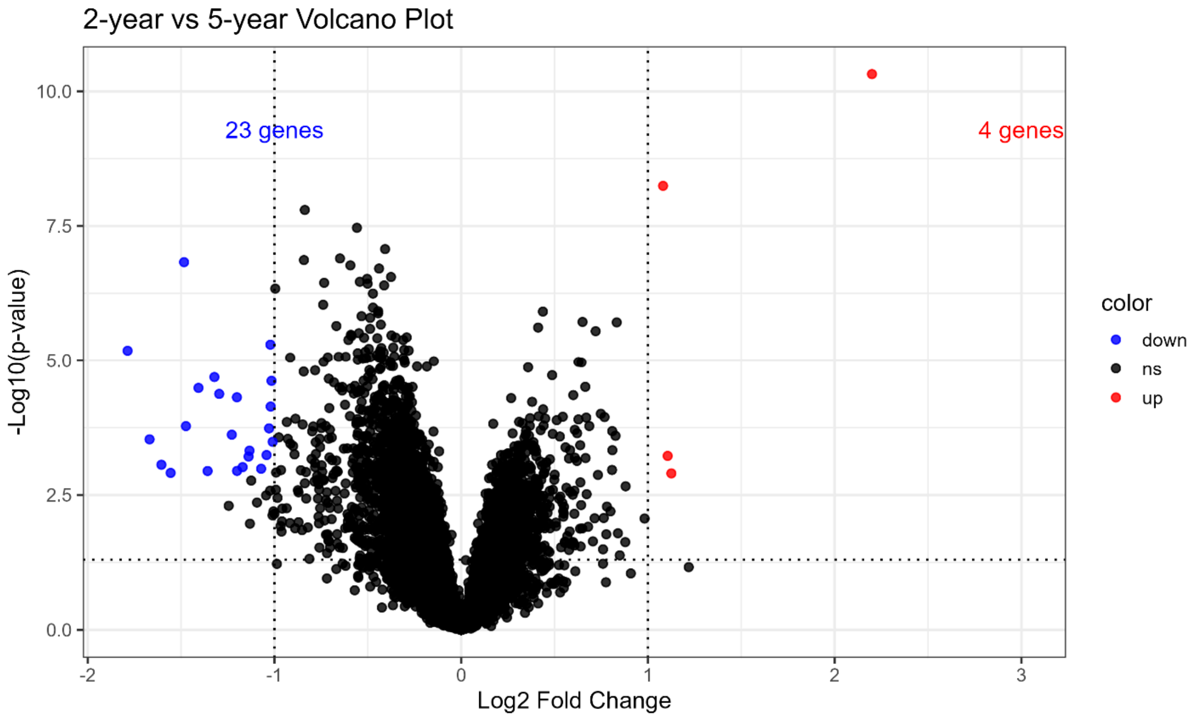

Supplementary Figure S2. Volcano plot of differential gene expression between 2-year and 5-year post-surgery

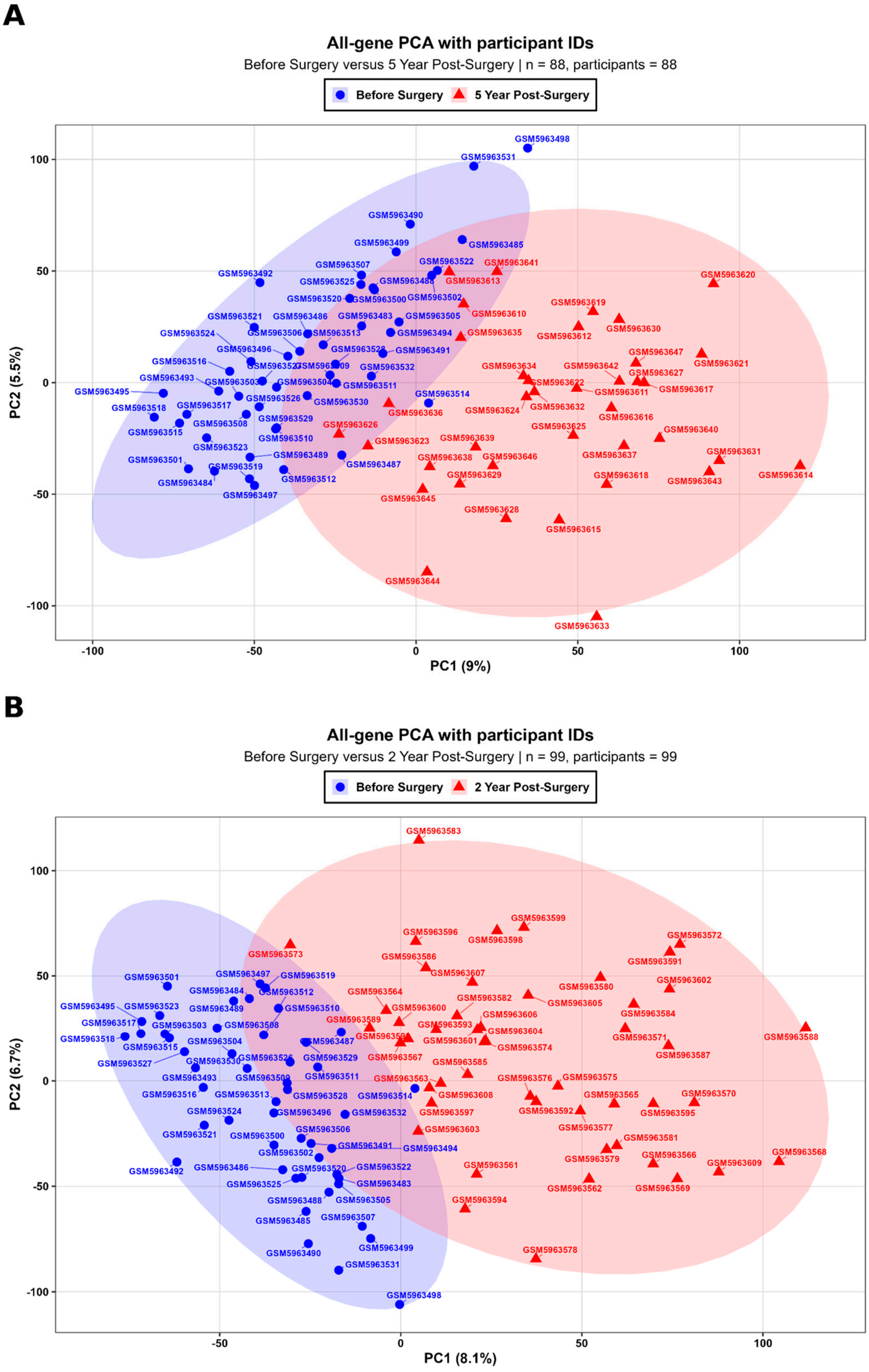

Supplementary Figure S3. All-gene principal component analysis of transcriptomic profiles before surgery and after surgery. (A) PCA comparison between before-surgery and 5-year post-surgery samples. (B) PCA comparison between before-surgery and 2-year post-surgery samples.

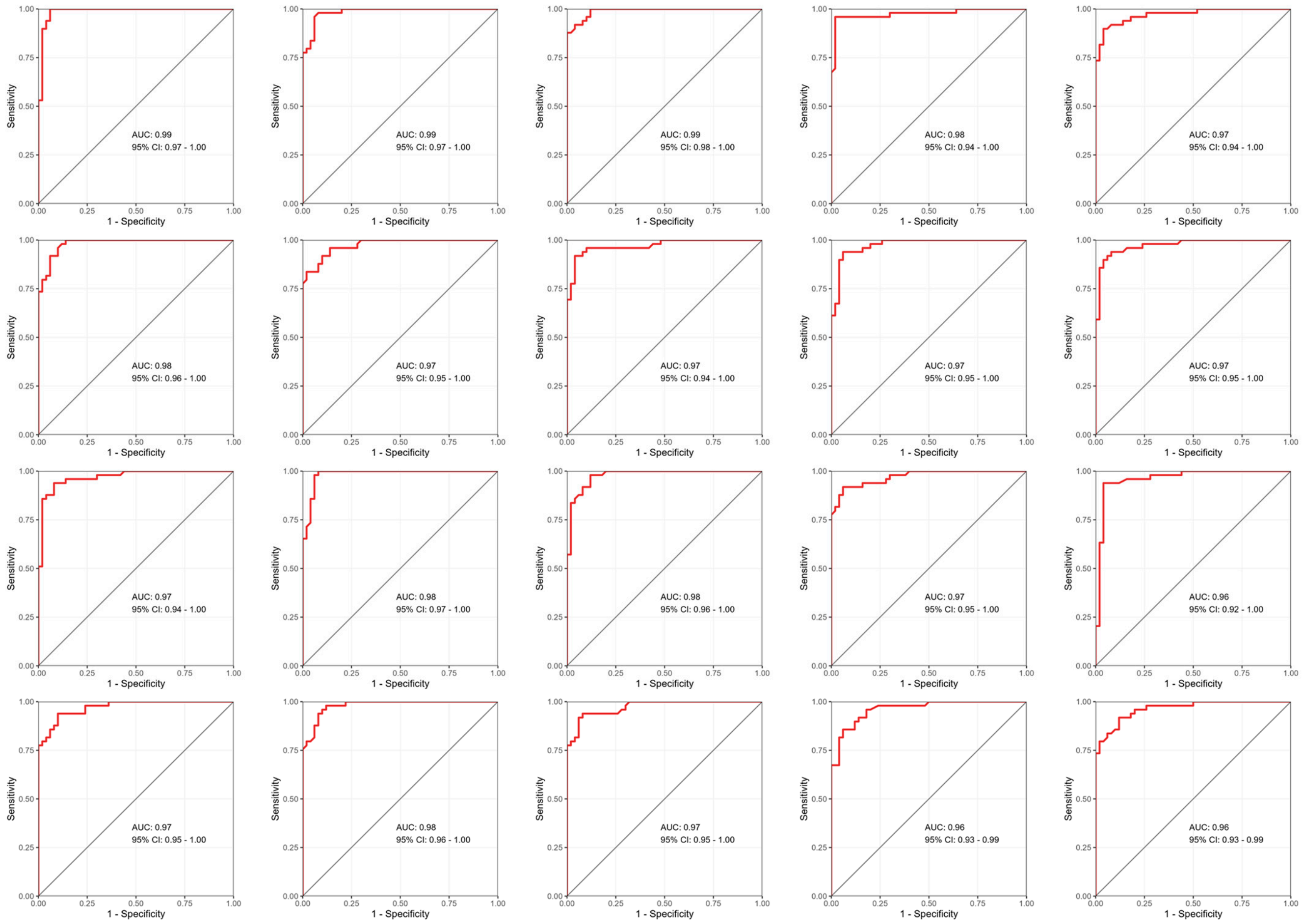

Supplementary Figure S4. Receiver operating characteristic (ROC) curves of the top discriminative genes distinguishing before-surgery and 2-year post-surgery samples.

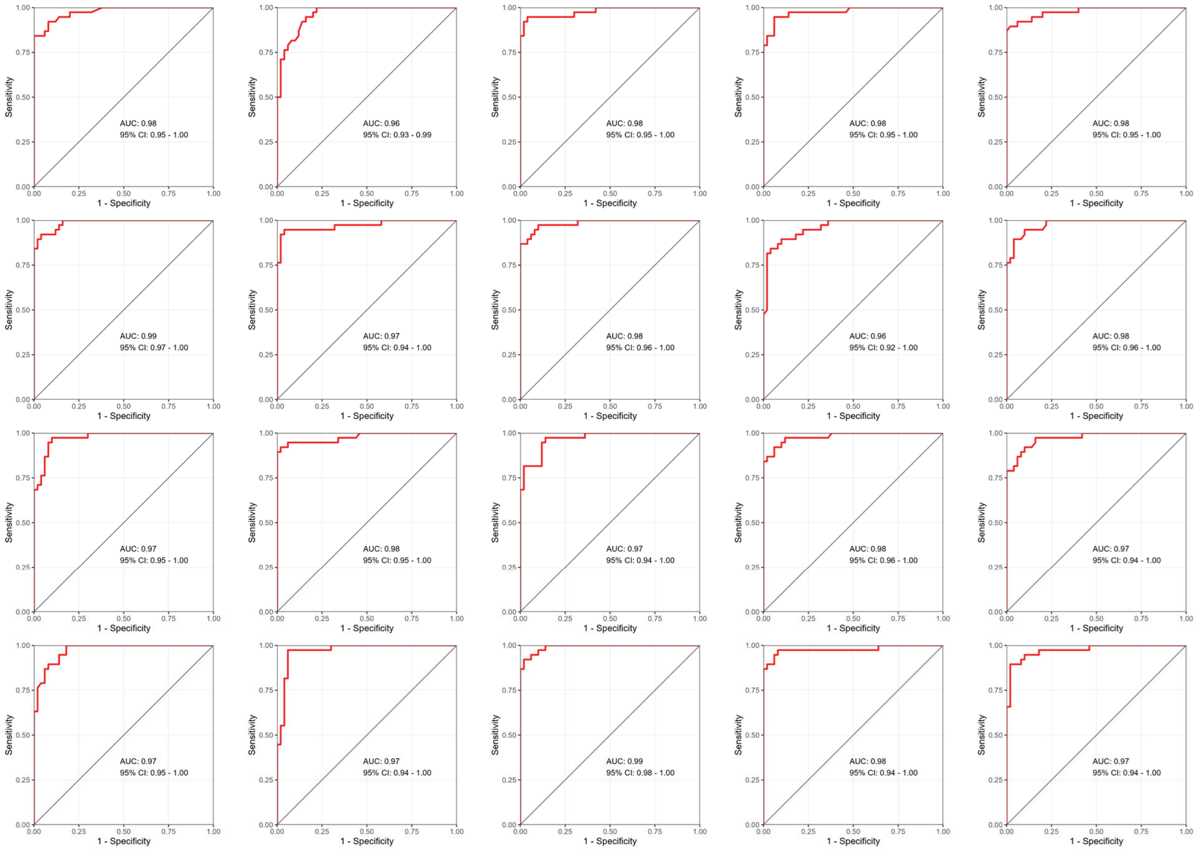

Supplementary Figure S5. Receiver operating characteristic (ROC) curves of the top discriminative genes distinguishing before-surgery and 5-year post-surgery samples.

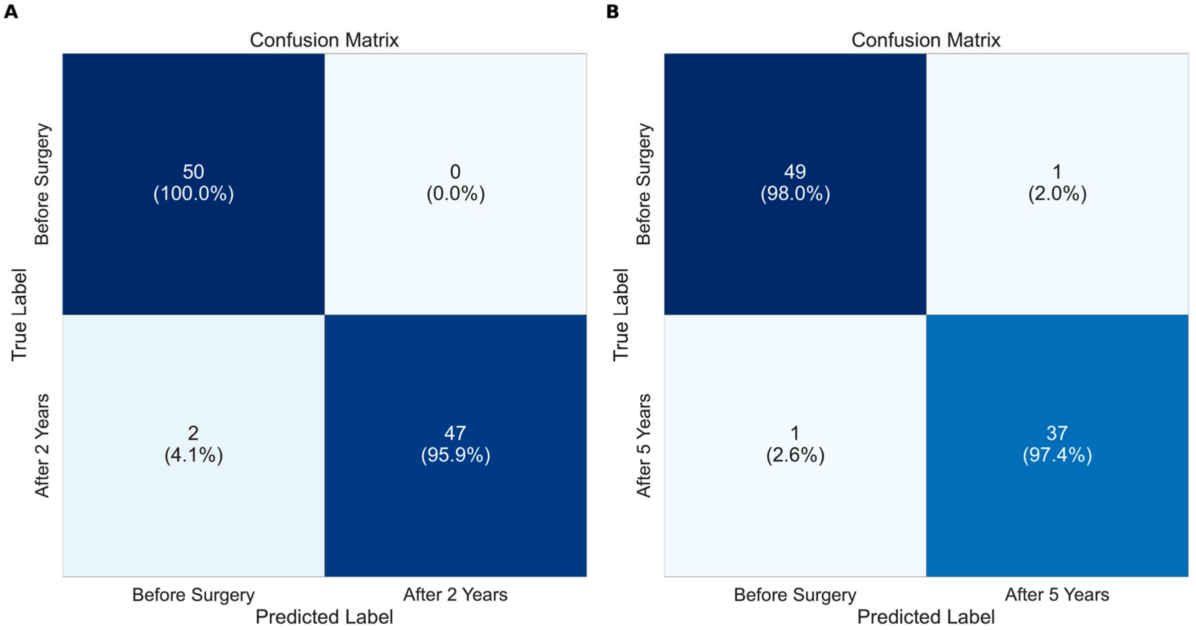

Supplementary Figure S6. Confusion matrices for the best-performing model (Random Forest) classifying (A) Before Surgery vs. 2 Years Post-Surgery and (B) Before Surgery vs. 5 Years Post-Surgery.

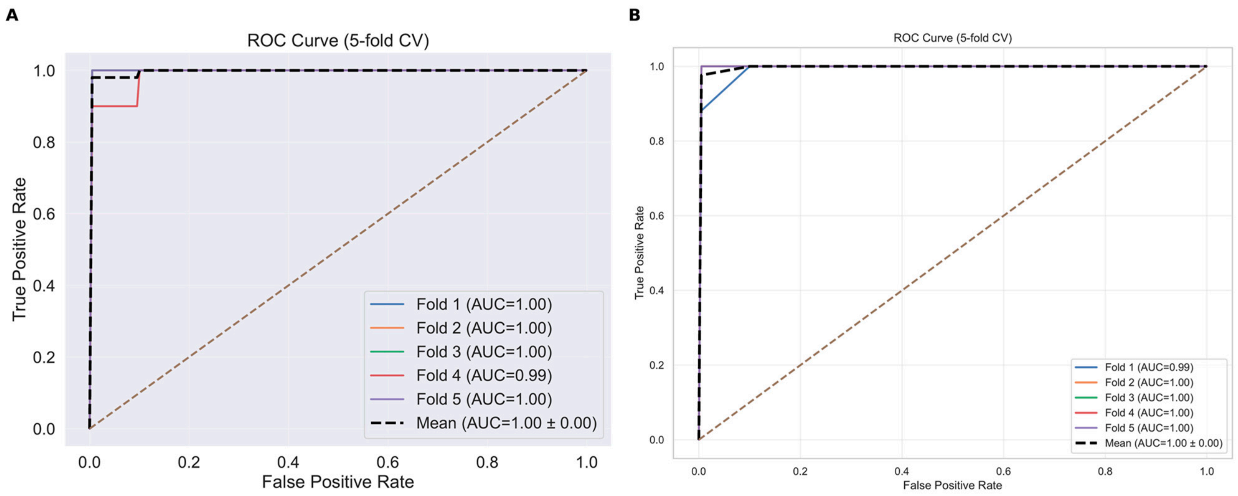

Supplementary Figure S7. ROC curves (5-fold cross-validation) for the best-performing model (Random Forest) classifying (A) Before Surgery vs. 2 Years Post-Surgery and (B) Before Surgery vs. 5 Years Post-Surgery.

Supplementary Section S1: Evaluation Metrics

The five evaluation metrics—weighted recall (sensitivity), specificity, precision, accuracy, and F1 score-are measured, which were mathematically defined in Equations (S1)–(S5).

$$\text{Accuracy}_{\text{class}_j} = \frac{TP_j+TN_j}{TP_j+TN_j+FP_j+FN_j} \quad \text{(S1)}$$

$$\text{Precision}_{\text{class}_j} = \frac{TP_j}{TP_j+FP_j} \quad \text{(S2)}$$

$$\text{Recall}_{\text{class}_j} = \frac{TP_j}{TP_j+FN_j} \quad \text{(S3)}$$

$$\text{F1\_score}_{\text{class}_j} = 2 \times \frac{\text{Precision}_j \times \text{Recall}_j}{\text{Precision}_j + \text{Recall}_j} \quad \text{(S4)}$$

$$\text{Specificity}_{\text{class}_j} = \frac{TN_j}{TN_j+FP_j} \quad \text{(S5)}$$

Supplementary Table S1: Integrated Machine Learning Feature Importance and Statistical Significance of Differentially Expressed Genes for Top ranked genes.

Before Surgery Vs After 2 Years					Before Surgery Vs After 5 Years				
feature	Mean_importance	log2FC	adj_p_value	AUC	feature	Mean_importance	log2FC	adj_p_value	AUC
BMP2K	0.111356	-1.39312	6.80E-24	0.975306	FCGR2A	0.125233	-3.6626	2.53E-16	0.978421
IL1RN	0.076156	-4.10502	4.64E-21	0.977347	NCKAP1L	0.064406	-2.8344	1.35E-18	0.972105
ACSS3.1	0.069492	2.229624	4.25E-26	0.987347	BTK	0.04743	-1.46345	4.96E-19	0.994211
HMOX1	0.031021	-1.60829	1.97E-23	0.984286	RNU11	0.028273	2.248874	2.56E-22	0.97
PCDH18	0.014664	1.568122	5.53E-21	0.961224	NIPSNAP3A	0.020722	1.469537	2.22E-16	0.975526
UCHL1	0.011017	-3.47153	2.98E-17	0.960612	TIMP1	0.018431	-2.38146	2.88E-21	0.991053
MAPRE1	0.010348	-0.9102	1.89E-18	0.948776	ECHDC2	0.015649	1.204968	1.11E-17	0.975263
ALCAM	0.008885	-3.15541	1.00E-23	0.99	CDC42	0.012035	-0.38734	2.62E-12	0.888684
ACACB	0.008396	1.114461	1.20E-14	0.890612	CTSC	0.010523	-1.58273	1.25E-12	0.916053
ADH1B	0.008323	1.312612	2.08E-19	0.976327	TYROBP	0.010439	-3.89832	3.70E-17	0.978684
NAALAD2	0.007922	1.401249	5.85E-18	0.957551	SFRP4	0.010289	-3.34615	2.69E-13	0.936053
NLN	0.007634	-1.3166	1.00E-23	0.961633	CD53	0.009048	-0.38734	2.62E-12	0.888684
PSAP	0.007419	-0.93062	7.91E-23	0.973061	PIK3CG	0.008741	-1.42658	7.12E-15	0.930263
ACVR1C	0.007051	2.097682	2.95E-25	0.985102	FCGR3A	0.008254	-2.71308	2.32E-17	0.958947
SH3BGRL3	0.006844	-1.91778	4.13E-20	0.970612	CLEC7A	0.007789	-2.82745	9.88E-22	0.972105
FCER1G	0.006663	-2.81773	3.94E-21	0.979388	HCLS1	0.007718	-1.71583	5.70E-17	0.952105
ZNF326	0.006391	0.301461	0.005275	0.647347	HACD4	0.007583	-1.34714	2.28E-14	0.914737
ADHFE1	0.006038	1.30538	7.55E-20	0.962245	FCER1G	0.00723	-3.68877	9.55E-17	0.98
ASAH1	0.00587	-1.14086	9.10E-17	0.937143	ADHFE1	0.006614	1.230874	4.91E-16	0.960526
TYROBP	0.00561	-2.72792	1.37E-19	0.966531	PTPRC	0.006358	-2.56205	2.63E-16	0.946316

Supplementary Table S2. Best-performing hyperparameter configurations of the tree-based models used for feature selection.

Model	Parameter	Value
XGBoost	max_depth	4
XGBoost	learning_rate	0.2
XGBoost	reg_lambda	1
XGBoost	n_estimators	150
XGBoost	subsample	0.9
XGBoost	colsample_bytree	0.9
XGBoost	random_state	42

XGBoost	eval_metric	Log loss
Random Forest	n_estimators	50
Random Forest	max_depth	10
Random Forest	random_state	0
Random Forest	n_jobs	−1
ExtraTrees	random_state	42
ExtraTrees	n_jobs	−1

Supplementary Table S3. Within-participant directional concordance and trajectory correlation of ALCAM, MSR1, PLA2G7, and NPL expression changes from baseline to 2 and 5 years post-surgery (n = 37 participants with complete longitudinal data). All Spearman correlations were statistically significant ($p < 0.0001$).

Gene	n	% same direction as group (2Y)	% same direction as group (5Y)	% subjects same dir 2Y vs 5Y	Spearman ϱ
ALCAM	37	100.00%	100.00%	100.00%	0.834
MSR1	37	100.00%	97.30%	97.30%	0.855
PLA2G7	37	100.00%	100.00%	100.00%	0.836
NPL	37	94.60%	100.00%	94.60%	0.859

Supplementary Table S4. Hyperparameter configuration of the final best-performing Random Forest classifier.

Model	Parameter	Value
	bootstrap	TRUE
	class_weight	None
	criterion	Gini
	max_depth	10
	max_features	sqrt
	max_leaf_nodes	None
	min_impurity_decrease	0
	min_samples_leaf	1
	min_samples_split	2
	min_weight_fraction_leaf	0
Random Forest classifier	n_estimators	100

	n_jobs	None
	oob_score	FALSE
	random_state	0
	verbose	0
	warm_start	FALSE
